# Phylogenomics of Globally Spread Clonal Groups 14 and 15 of Klebsiella pneumoniae

**DOI:** 10.1128/spectrum.03395-22

**Published:** 2023-04-26

**Authors:** Carla Rodrigues, Val F. Lanza, Luísa Peixe, Teresa M. Coque, Ângela Novais

**Affiliations:** a UCIBIO, Applied Molecular Biosciences Unit, Department of Biological Sciences, Laboratory of Microbiology, Faculty of Pharmacy, University of Porto, Porto, Portugal; b Unidad de Genómica Traslacional Hospital Universitario Ramón y Cajal (IRYCIS), Madrid, Spain; c CIBER en Enfermedades Infecciosas (CIBERINFEC), Madrid, Spain; d Associate Laboratory i4HB—Institute for Health and Bioeconomy, Faculty of Pharmacy, University of Porto, Porto, Portugal; e Servicio de Microbiología, Hospital Universitario Ramón y Cajal (IRYCIS), Madrid, Spain; University of Sydney

**Keywords:** IncF plasmids, accessory genome, antimicrobial resistance, Col plasmids, genomic epidemiology, high-risk clones, mosaic plasmids, multireplicon plasmids, plasmidome, resistome

## Abstract

Klebsiella pneumoniae sequence type 14 (ST14) and ST15 caused outbreaks of CTX-M-15 and/or carbapenemase producers worldwide, but their phylogeny and global dynamics remain unclear. We clarified the evolution of K. pneumoniae clonal group 14 (CG14) and CG15 by analyzing the capsular locus (KL), resistome, virulome, and plasmidome of public genomes (*n* = 481) and *de novo* sequences (*n* = 9) representing main sublineages circulating in Portugal. CG14 and CG15 evolved independently within 6 main subclades defined according to the KL and the accessory genome. The CG14 (*n* = 65) clade was structured in two large monophyletic subclades, CG14-I (KL2, 86%) and CG14-II (KL16, 14%), whose emergences were dated to 1932 and 1911, respectively. Genes encoding extended-spectrum β-lactamase (ESBL), AmpC, and/or carbapenemases were mostly observed in CG14-I (71% versus 22%). CG15 clade (*n* = 170) was segregated into subclades CG15-IA (KL19/KL106, 9%), CG15-IB (variable KL types, 6%), CG15-IIA (KL24, 43%) and CG15-IIB (KL112, 37%). Most CG15 genomes carried specific GyrA and ParC mutations and emerged from a common ancestor in 1989. CTX-M-15 was especially prevalent in CG15 (68% CG15 versus 38% CG14) and in CG15-IIB (92%). Plasmidome analysis revealed 27 predominant plasmid groups (PG), including particularly pervasive and recombinant F-type (*n* = 10), Col (*n* = 10), and new plasmid types. While *bla*_CTX-M-15_ was acquired multiple times by a high diversity of F-type mosaic plasmids, other antibiotic resistance genes (ARGs) were dispersed by IncL (*bla*_OXA-48_) or IncC (*bla*_CMY/TEM-24_) plasmids. We first demonstrate an independent evolutionary trajectory for CG15 and CG14 and how the acquisition of specific KL, quinolone-resistance determining region (QRDR) mutations (CG15), and ARGs in highly recombinant plasmids could have shaped the expansion and diversification of particular subclades (CG14-I and CG15-IIA/IIB).

**IMPORTANCE** Klebsiella pneumoniae represents a major threat in the burden of antibiotic resistance (ABR). Available studies to explain the origin, the diversity, and the evolution of certain ABR K. pneumoniae populations have mainly been focused on a few clonal groups (CGs) using phylogenetic analysis of the core genome, the accessory genome being overlooked. Here, we provide unique insights into the phylogenetic evolution of CG14 and CG15, two poorly characterized CGs which have contributed to the global dissemination of genes responsible for resistance to first-line antibiotics such as β-lactams. Our results point out an independent evolution of these two CGs and highlight the existence of different subclades structured by the capsular type and the accessory genome. Moreover, the contribution of a turbulent flux of plasmids (especially multireplicon F type and Col) and adaptive traits (antibiotic resistance and metal tolerance genes) to the pangenome reflect the exposure and adaptation of K. pneumoniae under different selective pressures.

## INTRODUCTION

Klebsiella pneumoniae subsp. *pneumoniae* (here referred to as K. pneumoniae) is currently considered one of the urgent threats for the emergence and spread of antibiotic resistance (ABR) (1, 2) and one of the top six human pathogens causing infections with high mortality rates (3). The increase of nosocomial infections caused by multidrug-resistant (MDR) K. pneumoniae and community-acquired infections caused by hypervirulent strains represents a major public health problem (4, 5). Recent advances in the population structure of K. pneumoniae using core-genome multilocus sequence typing (cgMLST) or comparative analysis of whole-genome sequences (WGS) revealed the predominance of a few clonal groups (CGs) associated with ABR, namely, CG15, CG29, CG147, CG101, CG231, CG258, and CG307 (4, 6). However, comprehensive genomic analyses are available for only a few of these predominant CGs (CG258, CG307, CG101, and CG147) (7–10).

Strains belonging to K. pneumoniae sequence type 14 (ST14) and ST15 represent 2.1% and 5.2% of the publicly available genomes (accessed from the GenBank assembly repository on July 2020), respectively, and are frequently producers of extended-spectrum β-lactamases (ESBL) or carbapenemases that confer resistance to different antibiotics and are involved in hospital outbreaks worldwide (11, 12). ST15 and ST14 were initially considered to be highly related because all MLST alleles are identical except one point mutation in *infB* (https://bigsdb.pasteur.fr/klebsiella/) (13, 14). Further analyses of disparate genome data sets (8 to 94 genomes; median = 23) led to variable conclusions, most considering CG15 and CG14 to be two different CGs (6, 15–17). Furthermore, the apparent frequent recombination events illustrated by the diversity of capsular types (KL) within ST15 and ST14 lineages circulating in Portugal since at least 2010 (18–21) and available recombination-free maximum likelihood phylogenetic inferences reinforce the need for a comprehensive phylogenetic analysis (15).

Available studies suggest that ST15 and ST14, as well as other MDR K. pneumoniae STs, are deep-branching lineages with a low nucleotide divergence (<0.5%) but highly variable accessory genomes (15). Such genomic variation is apparently due to large homologous recombination events involving the capsule locus or via exchange of plasmids, phages, or integrative conjugative elements (ICEs) (4, 15). However, few studies have provided detailed descriptions of the accessory genome, and especially of the plasmidome, in order to explain the adaptation and diversification of these K. pneumoniae CGs (16, 22). Furthermore, most studies have addressed plasmid diversity by replicon typing (23), which fails to establish whole plasmid entities. Technical limitations for assembling and sorting short-read sequences and the cost of long-read sequencing have limited the number of high-resolution analyses in large collections of isolates (24–27) until recently (28, 29).

This study analyzed the diversity and evolution of CG14 and CG15 K. pneumoniae genomes focusing on both its core and accessory genomes (antimicrobial resistance, capsule polysaccharide locus, and plasmidome) using high-resolution bioinformatic tools (30, 31). A detailed description of the plasmidome is also provided.

## RESULTS

### Phylogenomic analysis of global K. pneumoniae CG15 and CG14.

We performed a detailed phylogenomic analysis of 235 nonduplicated CG14 (*n* = 65; 63 ST14 and 2 single-locus variants [SLV]) and CG15 (*n* = 170; 162 ST15 and 8 SLV) isolates. These included seven out of the nine *de novo*-sequenced genomes (5 ST15 and 2 ST14) representing the five major lineages circulating in Portugal since early 2000s (Table 1). They have been previously categorized by diverse typing methods, such as multilocus sequence typing (MLST), pulsed-field gel electrophoresis (PFGE), and Fourier transform infrared (FT-IR) spectroscopy, that together supported lineage definition (18–21). These were substantiated with publicly available genomes (*n* = 228; 63 CG14 and 165 CG15) that met our inclusion criteria, as described in Materials and Methods. They correspond to strains isolated mainly from humans (97%) in Europe (55%), Western and Southeastern Asia (16%), and North America (7%) between 1980 and 2018 (see Fig. S1 in the supplemental material).

**TABLE 1:** K. pneumoniae isolates sequenced in this study and genome sequence information^*a*^

Strain	Isolation yr	ST	KL type	No. of contigs	Sequencing coverage	Total length (bp)	GC (%)	*N*_50_	Largest contig (bp)	Accession no.	Reference(s)
H49	2003	14	2	78	104	5,590,357	57.32	350,404	701,584	GCA_013504725.1	19, 21
H1122	2010	14	16	75	105	5,576,295	57.21	399,091	1,013,553	GCA_013504725.1	18, 19
44	2013	15	24	109	106	5,745,985	56.95	210,873	584,875	GCA_013620905.1	19
C1686*	2012	15	24	130	112	6,050,103	56.46	210,874	472,869	GCA_013521185.1	19
C1693	2012	15	24	132	105	5,649,365	57.12	184,651	371,151	GCA_013620925.1	19
H1119	2010	15	39	115	102	5,529,166	57.26	162,787	429,762	GCA_011090325.1	18, 19
C1699	2012	15	112	108	103	5,580,881	57.05	220,754	472,869	GCA_011065465.1	19
C1694*	2012	15	19	90	101	5,453,399	57.17	203,303	584,887	GCA_013620895.1	19
K47	2012	15	110	115	106	5,734,620	56.63	204,524	584,887	GCA_013620875.1	19, 79

a All strains were of phylogenetic group Kp1 and type O1. ST, sequence type; KL, capsular locus. Asterisks indicate strains that were closely related to strain 44 (shared <21 SNPs) and were excluded to avoid overrepresentation in the final data set (see Materials and Methods for details).

**FIG 1:**
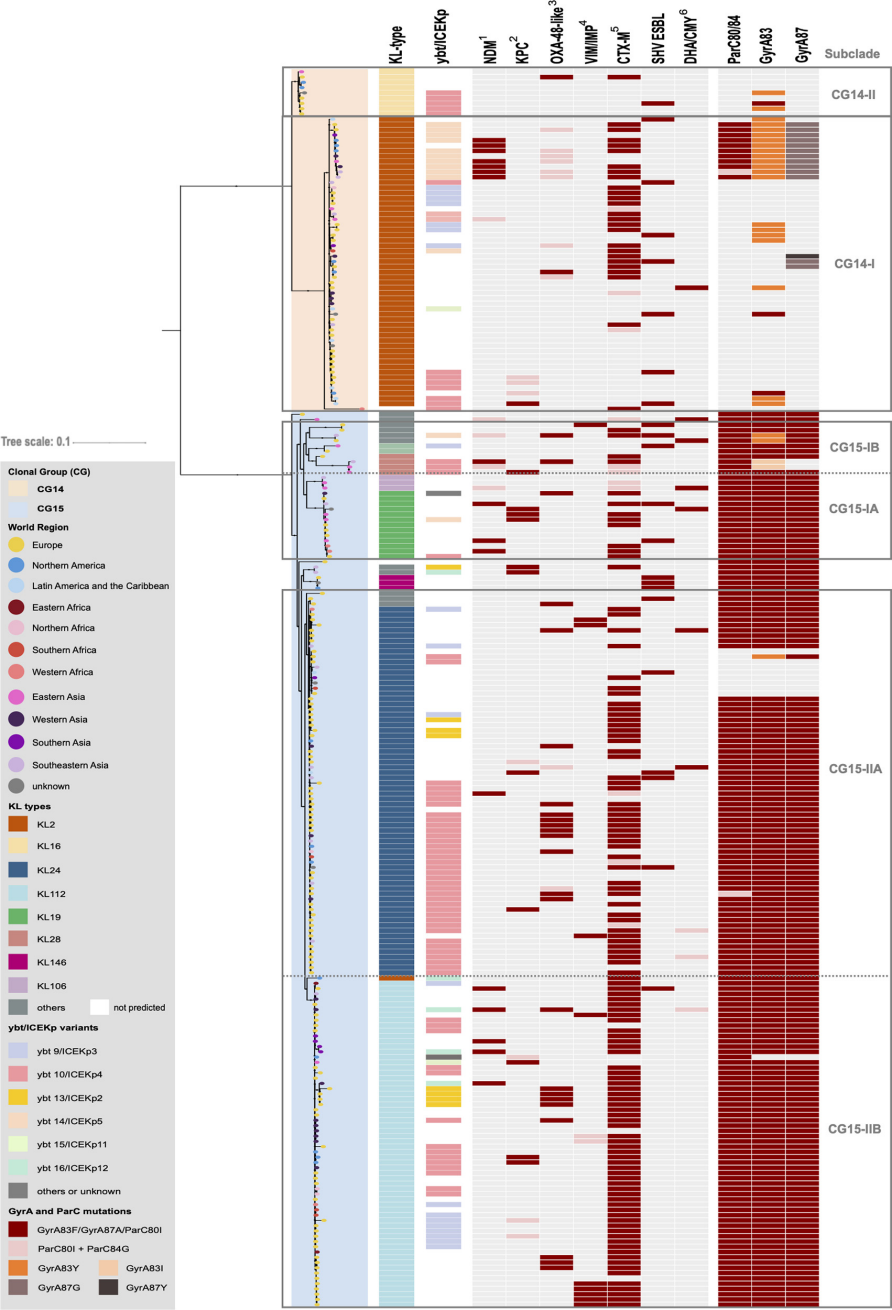
Phylogenetic structure of K. pneumoniae CG14 and CG15. Shown is a maximum likelihood tree (model GTR+F+ASC+R4) inferred from 35,783 SNPs extracted from the alignment of 4,420 core genes and rooted using two outgroups (K. pneumoniae ST540 Kpn0019 [SRA accession number SRR2098710] and K. pneumoniae ST101 Kp_Goe_33208 [GenBank assembly accession number GCF_001902435.1]). Branch lengths represent the number of nucleotide substitutions per site (scale, 0.01 substitution per site). The two main clades correspond to CG14 and CG15 and are shaded accordingly (see key), and main subclades thereof are delimited within rectangles. The branch tips are colored by the world region of isolation (see key). Capsular locus (KL) type and yersiniabactin-carrying ICEKp are colored according to their variants (see key). Acquired β-lactamases and GyrA and ParC mutations are indicated by colored rectangles when present, the different variants represented by colors according to the key. 1, dark pink indicates NDM-1 and light pink indicates other NDM variants; 2, dark pink indicates KPC-2 and light pink indicates KPC-3; 3, dark pink indicates OXA-48 and light pink indicates other OXA-48-like variants; 4, dark pink indicates VIM and light pink stands for IMP; 5, dark pink indicates CTX-M-15 and light pink indicates other CTX-M variants; 6, dark pink indicates DHA-1 and light pink stands for CMY.

Figure 1 shows the maximum-likelihood phylogenetic tree based on the concatenation of 4,420 core genes (representing ~80% of a K. pneumoniae genome) which grouped the genomes in two large clades corresponding to the CG, and six main subclades (2 CG14 and 4 CG15) that were in accordance with the capsular type (KL). The CG14 clade is split into CG14-I (KL2, *n* = 56) and CG14-II (KL16, *n* = 9), whereas CG15 is structured into CG15-I and CG15-II, which are further subdivided into two subclades arbitrarily designed “A” and “B,” as follows: CG15-IA (mainly KL19, *n* = 16), CG15-IB (variable KL types, *n* = 10), CG15-IIA (mainly KL24, *n* = 73), and CG15-IIB (KL112, *n* = 63). The single nucleotide polymorphism (SNP) median distance between CG14 and CG15 genomes was 2,452 SNPs/megabase (ranging between 7,500 and 12,143 SNPs) but much lower within CG14 (541 to 2,328 SNPs/megabase) or CG15 (252 to 1,084 SNPs/megabase) (Fig. S2).

In contrast with the correlation of particular KL types with the CG14 and CG15 subclades, virulence-factors (VF) appear to be widely distributed throughout the phylogeny (Table S1). Ninety-two percent of the genomes carried the O1 antigen type (92% of these are variant O1v1), while the occurrence of other O-antigen types was sporadic (3 O2 and 1 O4). All CG15 and CG14 genomes carried the *kfu* operon, encoding an iron ABC transporter, and a complete copy or remnants of the *kpi* chaperone-usher pilus system, the latter associated not only with CG15 as previously reported (32) but also with CG14. Eight different lineages of the yersiniabactin siderophore cluster (*ybt*) associated with eight different ICEKp structures and 48 *ybt* locus sequence types (YbSTs) were detected in 51% (120/235) of the genomes distributed throughout the phylogeny (Fig. 1). The highest diversity of *ybt* lineages was identified in subclades CG15-IIB (KL112) and CG14-I (KL2) (6 and 5 sublineages, respectively). The lineage *ybt*10/ICEKp4 was the most frequently detected (57% [68/120]), being overrepresented in the CG14-II (56%, 5/9) and CG15-IIA (51%, 37/73) subclades, respectively. The second most common (17% [20/120]) was the *ybt*9/ICEKp3 lineage, detected more frequently in CG15-IIB (14% [9/63]) and CG14-I (13% [7/56]). Minority lineages were *ybt*14/ICEKp5 and *ybt*13/ICEKp2, the former detected in 20% of the CG14-I genomes (Fig. 1 and Table S1). Only one strain (ST2174-KL2, SLV ST14) contained the putative virulence genes *rmpA2*, *iuc*, and *iro*, which encode a mucoid phenotype regulator and aerobactin and salmochelin siderophores, respectively. Three other ST15 genomes (2 KL112 and 1 KL24) carried the *clb* locus (encoding the genotoxin colibactin), *iuc*, and/or *rmpA2*.

### Phylogenetic structure and diversity within K. pneumoniae CG14.

The 65 CG14 genomes are grouped into two deeply branched subclades of variable size and diversity: CG14-I (86% KL2; 7 to 546 SNPs/megabase) and CG14-II (14% KL16; 29 to 108 SNPs/megabase) (Fig. 1 and Table S2). A time-scaled phylogenetic inference (root-to-tip regression analysis: *R*^2^ = 0.1474 [Fig. S3]) suggested a deep-branching structure of these two CG14 subclades and an evolutionary rate within CG14 of 4.76 × 10^−7^ substitutions/site/year (95% highest posterior density [HPD], 3.30 × 10^−7^ to 6.21 × 10^−7^) (Fig. S4). According to this prediction, the emergence of the CG14 ancestor was estimated to be around 1904 (95% HPD, 1862 to 1941), and those of the CG14-I and CG14-II subclades were estimated to be around 1932 (95% HPD, 1904 to 1955) and 1911 (95% HPD, 1865 to 1952), respectively.

K. pneumoniae CG14 isolates were identified in 26 countries (45% in Europe, 27% in Asia, and 21% in America) between 1980 and 2018 (Fig. S1). One K. pneumoniae ST14-KL2 genome from Portugal dates back to 1980 and carries only *strAB* genes, while Portuguese genomes from the early 2000s (*n* = 4; 2003 to 2014) represent isolates causing infections in hospitalized patients from different studies that contributed to the dissemination of genes encoding SHV-106 (ST14-KL16), TEM-24, or KPC-3 (ST14-KL2) (27, 28) (Table 1). Genes encoding ESBL (*bla*_CTX-M-15/-3/-9/-36_, *bla*_TEM-24_, or *bla*_SHV-12/-106_), AmpC (*bla*_DHA-1_), and/or carbapenemases (*bla*_OXA-232_, *bla*_NDM-1_, or *bla*_KPC-2/-3_) were observed in 65% of the CG14 genomes, and they were more frequently detected among CG14-I than among CG14-II isolates (71% versus 22%; *P = *0.0042). Of note, 11 highly related CG14-I genomes (39 to 259 SNPs; mean, 124 SNPs) carried multiple antibiotic resistance genes [*bla*_NDM-1_, *bla*_CTX-M-15_, *bla*_OXA-232_, *qnrB1*, *armA*, *aac(6′)-Ib-cr*, *aac(3)-IId*, *aph(3′)-VI*, *aadA2*, *dfrA1*, *dfrA12*, *catA1*, *catB4*, and/or *cmlA5*], specific mutations in quinolone resistance-determining regions (QRDR), and a specific mutation in *ompK36* (encoding an OmpK36GD variant) (33), showing dissemination of a pan-drug-resistant sublineage in 7 countries in Asia, the United States, and the United Kingdom between 2010 and 2016. Mutations in GyrA were uncommon and variable in CG14 genomes (6 to 19% 83Y, 83F, or 87G). *bla*_SHV-28_ was the most frequent narrow-spectrum chromosomal SHV β-lactamase gene identified (57%), but 7 other variants were also detected differing from *bla*_SHV-28_ by a 39-bp insertion (*bla*_SHV-100_) or by 5 to 9 SNP (all other variants most of which were *bla*_SHV-1_) (Table S1).

### Phylogenetic structure and diversity within K. pneumoniae CG15.

The 170 K. pneumoniae CG15 genomes were subdivided into two major subclades (CG15-I and CG15-II); each of these was further subdivided into two subclades, with all four identified putatively as CG15-IA (9% KL19 and KL106; 7 to 409 SNPs/megabase), CG15-IB (6% variable KL types; 10 to 777 SNPs/megabase), CG15-IIA (43% KL24; 6 to 299 SNPs/megabase), and CG15-IIB (37% KL112; 6 to 346 SNPs/megabase) (Fig. 1 and Table S2). Of note, strains corresponding to highly related genomes carrying KL106, KL110, or KL39 were isolated in neighboring countries and might correspond to local adaptive events (Table S1). The time-scaled phylogenetic inference (root-to-tip regression analysis: *R*^2^ = 0.3361 [Fig. S3]) estimates the emergence of the ancestor of CG15 around 1952 (95% HPD, 1929 to 1969), from which derived initially a few diverse CG15-KL24 genomes with wild-type *gyrA* and *parC*, and around 1989 (95% HPH, 1982 to 1993) a major branch including all CG15-I and CG15-II genomes containing a specific set of mutations in the QRDR, namely, GyrA83F, GyrA87A, and ParC80I (Fig. S5). The CG15-IA subclade (KL19) emerged around 1995 (95% HPD, 1990 to 1999), whereas both the majority of CG15-IIA (KL24) and all CG15-IIB (KL112) emerged from a common ancestor (1990; 95% HPD, 1985 to 1994) in 1993 (95% HPD, 1988 to 1998) and 1992 (95% HPD, 1987 to 1996), respectively. The identification of a small set of genomes carrying KL24 (*n* = 5) in CG15-IIB suggests recombination and further selection of the KL112 capsular type. The evolutionary rate within CG15 is estimated at 7.78 × 10^−7^ substitutions/site/year (95% HPD, 7.28 × 10^−7^ to 9.33 × 10^−7^).

The CG15 genomes corresponded to isolates identified between 1980 and 2017 in 32 countries, most of them collected in Europe (59% [100/170]) and in the framework of the EuSCAPE study (https://pathogen.watch/collections/all?searchText=euscape) (65% [65/100]). CTX-M-15 or CTX-M-15 and OXA-48 producers from Portugal and sequenced *de novo* in this study were closely related (from 61 to 187 SNPs) to public genomes from other countries in Europe and Western Africa (Table S2). Most CG15 genomes (87%) carried genes encoding ESBL, AmpC, and/or carbapenemases. *bla*_CTX-M-15_ was the predominant gene (68%) and significantly enriched in subclade CG15-II (64% in CG15-I versus 92% in CG15-II; *P = *0.0004). Variants of *bla*_CTX-M_ (*bla*_CTX-M-3_, *bla*_CTX-M-14_, *bla*_CTX-M-33_, *bla*_CTX-M-55_, *bla*_CTX-M-88_, and *bla*_CTX-M-199_), *bla*_SHV_ (*bla*_SHV-2_ and *bla*_SHV-12_), *bla*_AmpC_ (*bla*_DHA-1_, *bla*_CMY-6_, and *bla*_CMY-16_), or other *bla* genes (*bla*_OXA-1_, *bla*_OXA-10_, *bla*_VEB-5_, and *bla*_GES-11_) were also detected. Among carbapenemase producers (38%), *bla*_OXA-48-like_ genes were predominant (41%), followed by *bla*_KPC-2_ (17%), *bla*_NDM-1_ (16%), and *bla*_VIM-4_ (9%). A higher diversity of β-lactamase-encoding variants was observed in genomes from the CG15-I subclade. Variants of *mcr* (*mcr-1*, *mcr-3*, and *mcr-9*) were identified in five genomes, four of them from Asia. Mutations/disruptions in *ompK35* (26%) and/or *ompK36* (12%) were present throughout the CG15 phylogeny. In contrast with the case of CG14, *bla*_SHV-28_ was identified in most (91%) CG15 isolates, whereas other variants (*bla*_SHV-1_ and *bla*_SHV-11_) were only occasional (Table S1).

### The accessory genome of K. pneumoniae CG15 and CG14.

**FIG 2:**
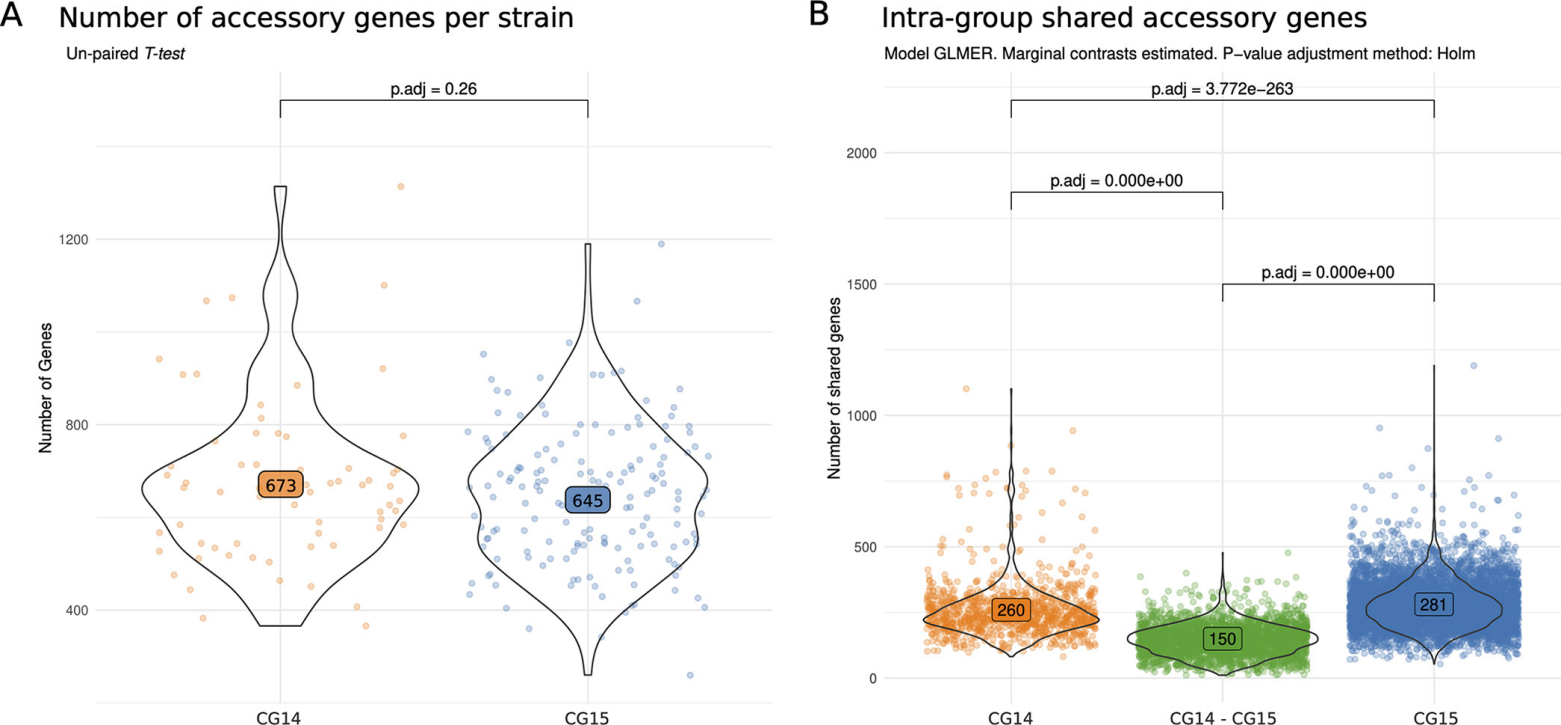
Statistical analysis of the accessory genome size and accessory gene sharedness between CG14 and CG15. (A) Average number of accessory genes in each CG; (B) accessory genes shared within CG14 or CG15 and between CG14 and CG15. Each set of shared genes between each genome was evaluated by correcting the model by the genomic distance between the genomes in order to mitigate the bias produced by sampling bias. The distribution was modeled with a GLME and a Poisson distribution.

We used the Pangenome Analysis Toolkit (PATO) to define and represent the size and composition of the accessory genomes of CG14 and CG15 (see Materials and Methods for details) (30). A total of 15,563 genes were identified as representing the accessory genome of both clonal groups (genes present in <80% of the strains). Despite the average numbers of accessory genes per strain being similar between CG14 (*n* = 673) and CG15 (*n* = 645) (*P* value = 0.26), the number of genes shared by the genomes of each CG (*n* = 260 in CG14 and *n* = 281 in CG15) is higher than that shared between the genomes of the two CGs (*n* = 150) (*P* value < 0.001) (Fig. 2), suggesting distinct accessory genomes for CG14 and CG15. The accessory genome network reveals that genomes from subclades CG14-I, CG14-II, CG15-IIA, and CG15-IIB have also a characteristic set of accessory genes, some of them shared between the groups (Fig. S6A). Nevertheless, the size of the common accessory genome in each subclade ranges from 274 and 329, in accordance with the core genome phylogenetic relatedness, and the differences between the subclades are significant (*P < *0.001) (Fig. S6B). A number of proteins (104 in CG14 and 117 in CG15) were enriched in each CG (>80% of the genomes; *P* value < 0.001); 39% and 61% of these, respectively, were annotated as hypothetical proteins. We also found variable numbers of enriched proteins the main subclades as follows: CG14-I, *n* = 97; CG15-IA, *n* = 81; CG15-IIA, *n* = 126; and CG15-IIB, *n* = 182. A high frequency (54%) of subclade enriched proteins were annotated as hypothetical proteins; the remaining were involved in carbohydrate metabolism or were ABC transporters, surface and outer membrane proteins, prophage integrases, toxins, and proteins associated with plasmid conjugation and partitioning. Of note, most (75%) highly specific proteins (*n* = 97; present in >80% of the subclade and in <30% of the total genomes) were observed in the CG14-I subclade (Table S3). Our results show that each CG (and its respective subclades) has its own accessory genome repertoire but with a great plasticity, as it can be deduced from the number of genes shared between CG.

### The plasmidome of K. pneumoniae CG15 and CG14.

**FIG 3:**
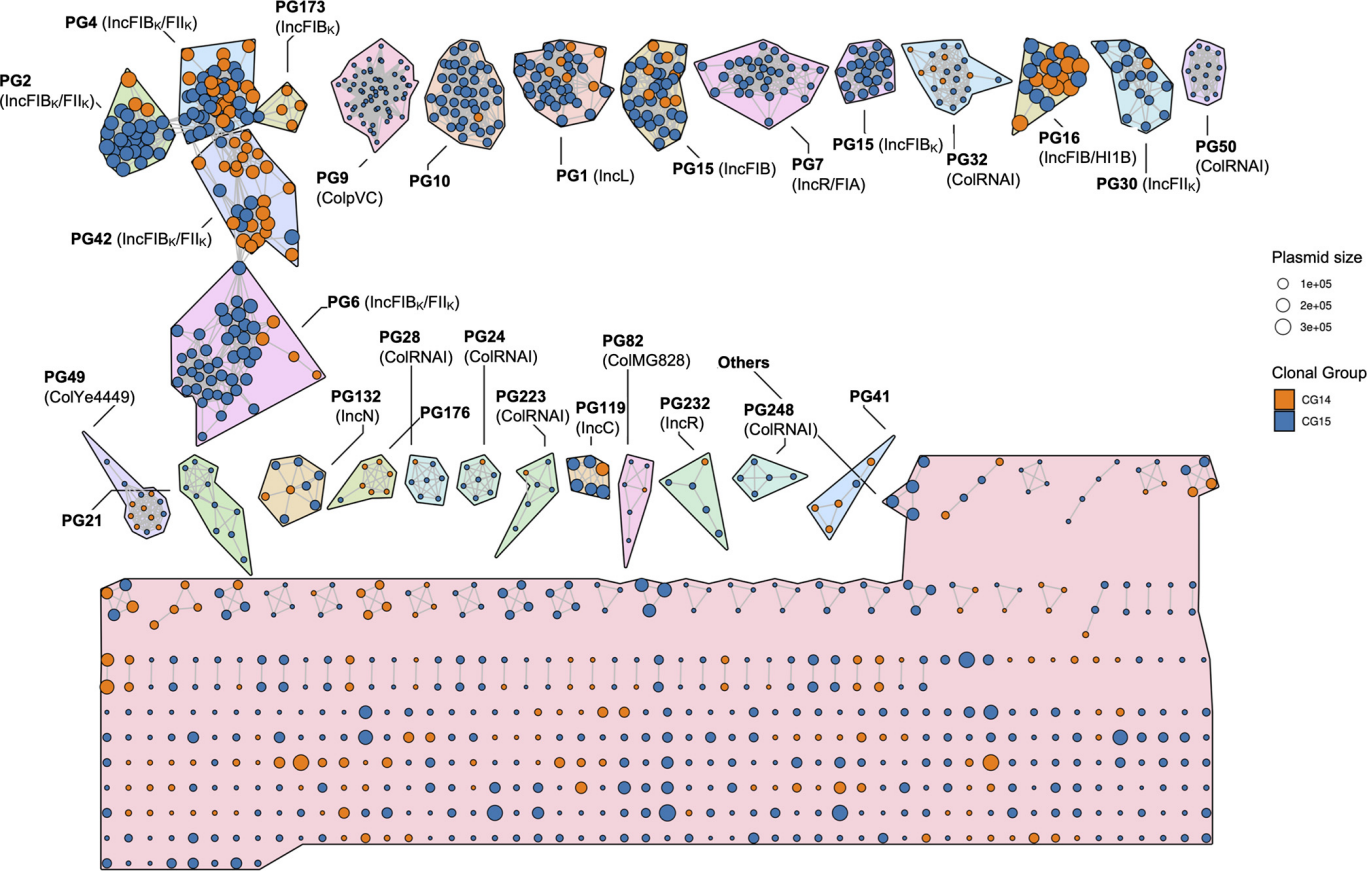
K-nearest neighbor network (K-NNN) of K. pneumoniae CG14 and CG15 plasmids. The network was built using the PATO k-nnn function. Each node represents one plasmid and is colored according to the CG. A group of similar plasmids (>5 plasmids) is represented by a plasmid group (PG), plasmid clusters being defined using the Louvain algorithm over the network structure. Each PG is arbitrarily designated with a number, and replicon content defined by MOB-typer is shown in parentheses (34). Differences in color shading between PGs is arbitrary. Each plasmid is connected with the 10 best hits if their Jaccard similarity is at least 0.5 and the difference in size is less than 50%.

Plasmid sequences were identified using MOB-Suite and plasmids were reconstructed from the assembly FASTA files using MOB-recon (34). Plasmid typing on the whole data set (replicon family, relaxase type, mate-pair formation type, and predicted transferability) was performed using MOB-typer (34) and PATO (30). Plasmids sharing at least 50% coverage/similarity were considered a “plasmid group” (PG); data on replication origin, relaxase type, antibiotic resistance genes (ARG), and metal tolerance genes (MTG) were collated to each PG (see Materials and Methods for details). We detected a total of 1,050 plasmids, of which 533 clustered in 27 PGs composed of >5 plasmids (from 5 to 59 plasmids), 184 corresponded to small PGs (from 2 to 4 plasmids), and 333 were singletons (Fig. 3).

**FIG 4:**
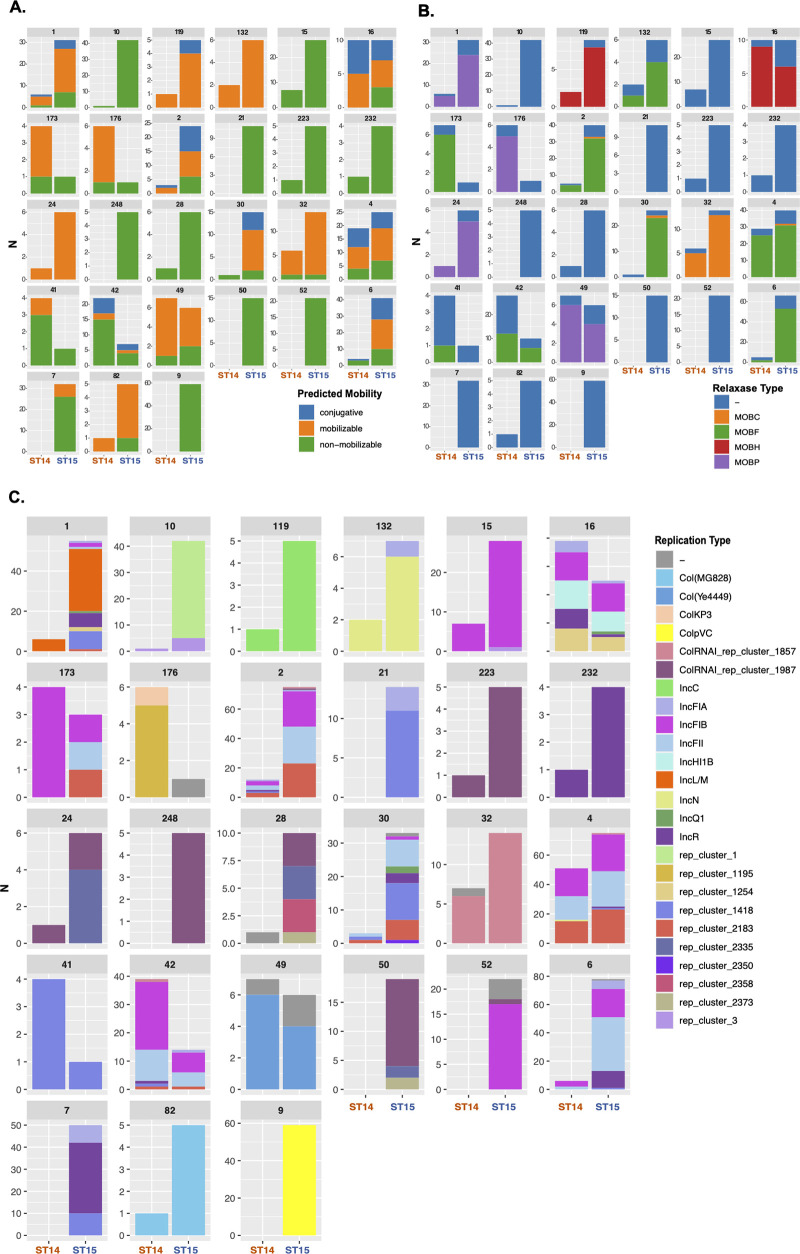
Predicted mobility and replicase and relaxase contents of each plasmid group (PG). (A) Distribution of plasmid predicted mobility within each PG in CG15 and CG14; (B) distribution of replicases (incompatibility group) within each PG in CG15 and CG14; (C) distribution of relaxases (MOB type) within each PG in CG15 and CG14.

The content of the 27 main PGs in replicons and relaxases, their predicted mobility, and their distribution in CG15 and CG14 are shown in Fig. 4. Replicons identified belonged to 9 known types (FII, FIA, FIB, L, HI1B, C, N, R, and Q1), 10 were untypeable (not classified by incompatibility typing schemes available), and 6 were Col types, which were observed alone or in variable combinations. They were part of 10 F-type PGs, 10 Col PGs, and 7 variable non-F-type PGs, 16 of which were abundant (present in >10 genomes). Twelve PGs carried no relaxase, whereas MOBF, MOBP, MOBH, and MOBC types were found, respectively, in 8, 4, 2, and 1 PG. Most plasmids were predicted as nonmobilizable (60%, based on the absence of conjugative machinery and/or relaxase) or mobilizable (32%, if conjugative machinery was absent), and a minority were predicted as conjugative (8%, when conjugative machinery was detected).

Ten PGs consisted of K. pneumoniae narrow-host-range F-type plasmids with multiple replicons, all except one highly abundant and distributed (from 16 to 45 genomes) (Fig. S7). They consisted of combinations of FII_K1/K4_ plus FIB_K_ with or without rep_2183 (50 to 300 kb; PG-2, -4, -6, and -42), FII_K1_+rep_1418+rep_2183 (60 to 175 kb; PG-30), FIB_pNDM-MAR_+HI1B_pNDM-MAR_+rep_1254 (214 to 340 kb; PG-16), R+FIA_HI1_ (17 to 70 kb; PG-7), and different FIB plasmids (50 to 162 kb; PG-15, -52, and -173), most of which (90%) were associated with ABR (see below) (Fig. 4 and Table S4).

Ten PGs corresponded to Col plasmids, most of them (70%) of broad host range (Fig. S7). Four of them were particularly frequent, namely, ColpVC (2 kb; PG-9), ColRNA_rep1857 (6 to 12 kb; PG-32), ColRNAI_rep1987 (4 kb; PG-50), and ColYe4449 (5 to 6 kb; PG-49). Other less frequent Col plasmids included ColMG828 (1.6 kb; PG-82) and other combinations of known and new Col types (*n* = 5 PGs) (Fig. 4C). It is noteworthy that most Col plasmids have been associated with plasmids from non-Klebsiella species: Salmonella enterica (ColpVC), Yersinia enterocolitica (ColYe4449), and Escherichia coli (4 Col plasmids, including ColMG828) (Table S4). The remaining PGs included the broad-host-range IncL (46 to 128 kb; PG-1 in 37 genomes), IncN (40 to 50 kb; PG-132 in 8 genomes), or IncC (160 to 180 kb; PG-119 in 6 genomes), the narrow-host-range IncR (PG-232), and new replication types, including rep_cluster1+rep_cluster3 (50 to 60 kb; PG-10) or rep_1418 (10 kb; PG-21 or 20 to 30 kb; PG-41), some of which were highly abundant (PG-10 in 43 genomes and PG-21 in 11 genomes) (Fig. 4C).

We detected an average number of PGs per genome of 4.65 (between 3 and 6), the number increasing from CG14 (average = 3.8) to CG15 (average = 4.98), a statistically significant difference (*P* value = 1.58e−4). Plasmids were unevenly distributed in the data set (243 in CG14 and 807 in CG15), some PGs being exclusively found in CG15 (PG-9, -10, -21, -50, and -248; 3 Col plasmids; 2 new plasmids). Thus, we used a hypergeometric test to identify PGs overrepresented in each CG (*P* value adjusted for multiple comparison < 0.05). We found that PG-4, PG-42, and PG-176 were overrepresented in CG14 and four PGs were overrepresented in subclades CG15-IIA and -B (PG-9 and PG-10; >80%) and CG15-IIB (PG-7 and PG-52; >80%). Only one of these was a multidrug resistance plasmid (Fig. S8).

### The acquired resistome and metalome of K. pneumoniae CG14 and CG15.

Fourteen out of the 27 PGs contained ARGs (Fig. S9) and nine of them also carried MTGs (Fig. S10). It is of note that they are plasmids of known IncF, IncC, IncL, IncN, and Col families, most ABR PGs (*n* = 10/14) carrying multiple resistance genes being multireplicon F plasmids. Four PGs carried only *bla*_TEM-1_, *bla*_OXA-232_, *tetA*, or *aph(3″)-Ib*+*aph(6)-Id*.

**FIG 5:**
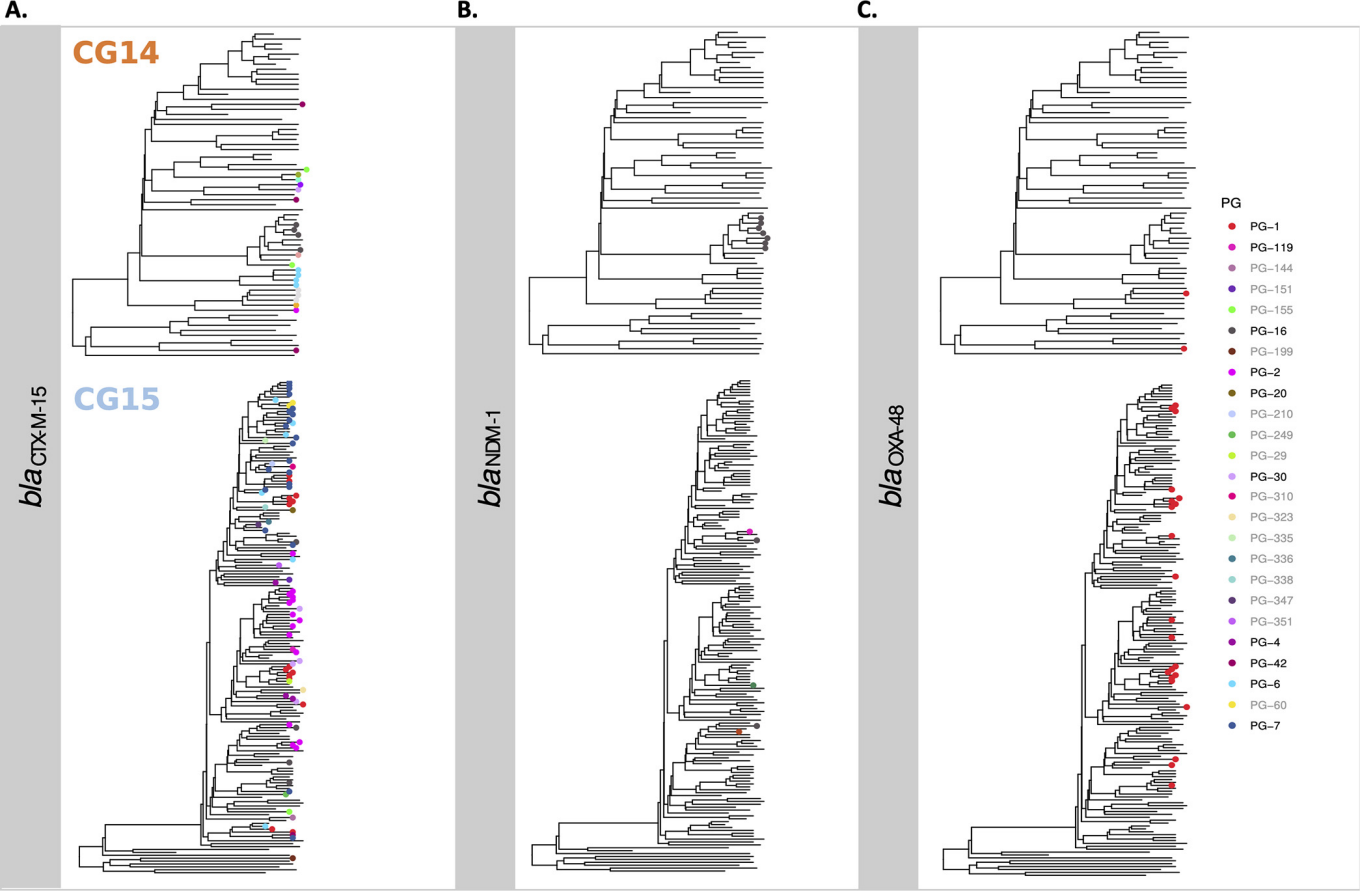
Distribution of plasmid groups carrying *bla*_CTX-M-15_, *bla*_NDM-1_, and *bla*_OXA-48_ across the phylogeny. Phylogenetic trees of each CG were obtained with BEAST (represented in Fig. S4 and S5). PGs carrying each β-lactamase gene were associated with each genome in the phylogenetic tree. In the key, black labels represent common PGs (>5 plasmids), and gray labels represent uncommon PGs (<5 plasmids).

The association of main ESBL and carbapenemase genes with particular PGs across the CG14 and CG15 phylogeny reflects the complex trajectories and acquisition routes of these genes among these CGs (Fig. 5). The *bla*_CTX-M-15_ gene was frequently linked to *bla*_TEM-1A_, *bla*_OXA-1_, *dfrA14*, *tetA*, *aac(6′)-Ib-cr*, *aph(6)-Id*, and *aph(3′)Ib* on a diversity of plasmid backgrounds (*n* = 9, consisting of variable combinations of FII_K1/K4_, FIB [FIB_K_, FIB_pNDM-MAR_, or FIB_pKPHS1_], HI1B, FIA_HI1_, R, or new replicons L and C) (Fig. S9), suggesting their location on a widespread transposable unit (35). Of note, this putative transposable unit was repeatedly acquired by variable mosaic F-type plasmids several times across the CG14 and CG15 phylogeny (Fig. 5A). Several of these are highly homologous to those of known hybrid plasmids of K. pneumoniae, such as pKPN307-D (GenBank accession number KY271407), pKP6402 (GenBank accession number AP018752), and pUUH239 (GenBank accession number CP002474), all derived from pKPN3 plasmid (GenBank accession number NC_009649 [Table S4]). *bla*_NDM-1_ was acquired once in CG14 by PG-16 (IncHI1B+FIB) and at least four times in CG15 by four different PGs (IncHI1B+FIB, IncC, or other), on some occasions together with *bla*_CTX-M-15_ (Fig. 5B). In contrast, *bla*_OXA-48_ was located only on IncL plasmids related to pOXA-48 (GenBank accession number JN626286), which was acquired multiple times throughout the CG15 phylogeny and less frequently in CG14 (Fig. 5C).

*bla*_OXA-232_ was exclusively associated with a hybrid Col-type PG-176 in closely related CG14-I (KL2) (Table S4). The *bla*_TEM-24_ or *bla*_CMY_ genes were located on IncC and the *bla*_SHV-12_ or *bla*_KPC-2_ genes on IncN, whereas *bla*_VIM_ and *bla*_KPC-3_ variants were occasionally detected in different PGs of 10 and 7 genomes, respectively. It is of interest that certain predominant PGs carry diverse ESBL or carbapenemase genes, namely, IncC (*bla*_CTX-M-15_, *bla*_NDM-1_, *bla*_SHV-5_, *bla*_CMY_, or *bla*_TEM-24_), IncN (*bla*_KPC-2_, *bla*_NDM-1_, or *bla*_SHV-12_) or the FIB-HI1B multireplicon plasmid (*bla*_CTX-M-15_, *bla*_NDM-1_, or *bla*_DHA-1_). PG-16 (FIB+HI1B) carried the highest number and diversity of ARG and showed regions identical to reference NDM-1-encoding plasmids such as pPMK1-NDM and pNDM-MAR (GenBank accession numbers CP008933 and JN420336, respectively).

MTG were mainly located on AMR-PGs (*n* = 8/9; 7 multireplicon F plasmids, 1 IncL plasmid, and 1 IncC plasmid). All multireplicon FII_K1/K4_+FIB_K_ plasmids (PG-2, -4, -6, and -42) were enriched in mercury (*mer*), copper (*pco*), silver (*sil*), and arsenic (*ars*) operon genes. Other F-type plasmids carried *mer*, *pco*, and *sil* (PG-16; FIB_pNDM-MAR_+HI1B), *pco* and *sil* operons (PG-52, FIB_K_) or only *merA* or *merE* genes (PG-7, FIA+R). IncN and IncC plasmids carried different *mer* operons (Fig. S10).

## DISCUSSION

This study revealed that the globally distributed K. pneumoniae CG14 and CG15 have independent evolutionary trajectories. Moreover, it provided the first comprehensive analysis of the plasmidome of global K. pneumoniae CGs showing the contribution of specific plasmids to the expansion of particular CGs and subclades.

The discrimination between CG14 and CG15 described here is also supported by another recent work that consider them as sublineage 15 (SL15) and SL14 according to a nomenclature based on cgMLST and life identification number (LIN) (17). According to this proposal, only the major CG14 subclades are differentiated in two distinct clonal groups (CG14-I as CG14 and CG14-II as CG10201), whereas CG15 subclades belong to the same clonal group (CG15). The deep-branching structure of their core genome shows a diversification that involves capsular switches and further expansion of the dominant subclades carrying specific KL types (KL2 in CG14 and KL24/KL112 in CG15). The most recent common ancestor of the major CG15 subclade (CG15-II) seems to have appeared in 1990s, contemporaneously to other globally distributed sequence types such as ST307 (1994; 95% HPD, 1974 to 2006), ST147 (KL64; 1994; 95% HPD, 1990 to 1998), ST101 (1989; 95% HPD, 1964 to 2007), and ST258 (1995; 95% HPD, 1988 to 1999) (7–10). Conversely, the common recent ancestor of major CG14 subclades seems to have emerged earlier (around 1911 or 1932), which is consistent with the differentiation and nomenclature proposed by Hennart et al. (17). Despite the differences in the predicted emergence dates of different CGs, the evolutionary rate of both CG14 and CG15 is lower (4.76 × 10^−7^ to 7.78 × 10^−7^ substitutions/site/year) than that observed for most global CGs (1.03 × 10^−6^ to 2.85 × 10^−6^ substitutions/site/year) but similar to that of hypervirulent ST23 (3.4 × 10^−7^ substitutions/site/year) (7–10). Nonetheless, these differences should be interpreted with caution since they might reflect the heterogeneity and variable robustness of the different data sets, especially those comprising relatively short timescales (36).

We found that CG and subclade differentiation can be explained by specific events involving the capsule and acquisition of mutations associated with fluoroquinolone resistance. Recombination events involving the capsule locus could have allowed the adaptation to human immunity and consequent selection and amplification of certain K. pneumoniae subpopulations (37–39), similarly to what has been described for Streptococcus pneumoniae or Neisseria meningitidis (40, 41). In fact, the recognition of these KL types as suitable evolutionary markers led to their use in diagnostics for categorizing K. pneumoniae isolates (including CG15 and CG14) in hospital surveillance and infection control programs using FT-IR spectroscopy (19, 42, 43). Besides the capsule, the acquisition of resistance to fluoroquinolones by most CG15 isolates through specific mutations in GyrA and ParC also mimics what has been reported for other contemporary high-risk clones of K. pneumoniae, Escherichia coli, Staphylococcus aureus, Clostridioides difficile, or Enterococcus faecium (7, 8, 44–47). In fact, fluoroquinolone resistance seems to be an advantage for the fitness of high-risk clones of different species (48, 49). Fluoroquinolone-resistant clones predominate in the elderly and people who often have a long history of exposure to health care centers, a risk factor for the acquisition of other antibiotic resistance genes (50, 51).

Though the sample analyzed is overrepresented by genomes from European countries, the detection of all CG14 and CG15 subclades in different continents and wide geographical areas reflects their global distribution (11, 12), similar to what is reported for CG147, CG258, CG307, or CG101. Most genomes carried genes encoding extended-spectrum β-lactamases, acquired AmpCs, and/or carbapenemases (81%), reflecting the sampling bias toward MDR strains from the nosocomial setting. The role of plasmids in the expansion of MDR K. pneumoniae CGs remains largely unexplored, probably due to the difficulty in analyzing plasmidomes and mobilomes in large genome data sets. Although a few recent studies have analyzed plasmids of a high number of genomes from *Enterobacterales* using large-scale network analysis (28, 29), this work is the first study using such an approach to characterize the plasmidome of particular K. pneumoniae CGs.

Our results revealed an overrepresentation of F and Col plasmids, whose distribution in other CGs is largely unknown. The high degree of mosaicism observed, especially on narrow-host-range multireplicon F-type plasmids, is in agreement with previous observations and is suggestive of the impact of recombination in the dissemination of ARG and/or K. pneumoniae adaptation and of a degree of specialization resulting from long-term plasmid-host coevolution (52–54). The high heterogeneity of small plasmids (essentially ColE1 type) long evolving in *Enterobacterales*, some of them particularly abundant and linked to CG15 (ColpVC and ColRNAI_rep1987), is also of note. Col plasmids have been implicated in extraintestinal virulence and/or gastrointestinal colonization in E. coli (55, 56). In K. pneumoniae, their role is still largely unexplored, although the association with adaptive traits such as ARG (e.g., *bla*_OXA-232_), cell metabolism, virulence, defense from phages, and heavy metal resistance, together with their variability and transposase content (IS*26*, IS*4321*, Tn*3*-like), suggests an important role in adaptation (26, 55, 57–59). The diversity of plasmids harboring replicons untypeable by classical plasmid typing schemes (23, 60) reinforces the need to improve current K. pneumoniae plasmid categorization approaches (34, 61). Cryptic plasmids (50 to 60 kb; PG-10 or 10 kb; PG-21; noncarriers of ABR genes) were common in a major CG15 subclade, suggesting a role for unknown features in adaptation. The high occurrence of nonconjugative/nonmobilizable plasmids is not surprising and reflects the high recombination of the mobile genetic elements within and/or between high-risk clones with frequent deletion and acquisition events (61). However, their mobilization is not compromised because of the association with helper entities (e.g., conjugative plasmids and ICEs) (52, 62, 63).

One of the most important observations is that only a few known PGs are responsible for the dissemination of ARG and MTG, specifically, multireplicon F plasmids and those of classical L, C, and N incompatibility groups. Of particular relevance is the high frequency of mosaic pKPN-3-like plasmids in CG15, previously observed in other CGs, which confers on them a significant role in the acquisition, spread, and persistence of contemporaneous ESBL and carbapenemase genes (*bla*_CTX-M-15_, *bla*_KPC_, and *bla*_NDM-1_) in K. pneumoniae (63–68). The acquisition of ARG and/or MTG by these plasmid backgrounds seems to have contributed to the diversification and expansion of CG15, particularly the GC15-IIB subclade (KL112). In fact, *bla*_CTX-M-15_ is the most frequently acquired *bla*_ESBL_ gene (38% in CG14 versus 68% in CG15; *P = *0.00006), increasing in prevalence from CG14-I (45%), CG15-IIA (62%), and CG15-IIB (92%). The repeated acquisition of the putative transposable unit containing *bla*_CTX-M-15_ by multiple F-type plasmids might have been key for this expansion, as reported for E. coli ST131 (69). It is also worth highlighting the high proportion of genes encoding acquired β-lactamases (71%; especially *bla*_CTX-M-15_, *bla*_OXA-232_, and/or *bla*_NDM-1_) identified in the CG14-I subclade, considering that KL2 is frequently associated with hypervirulent and antibiotic-susceptible K. pneumoniae (22). Our data reveal multiple trajectories for the dissemination of ARG across different plasmid and genetic backgrounds: (i) a single plasmid across multiple lineages (e.g., *bla*_OXA-48_-IncL), (ii) multiple plasmids across multiple lineages (e.g., *bla*_CTX-M-15_ or *bla*_NDM-1_ in multiple plasmids across the phylogeny), and (iii) multiple plasmids in one lineage (e.g., *bla*_CTX-M-15_ in plasmids from CG15-IIB). By providing opportunities for plasmid shuffling and evolution, these trajectories contribute to the expansion and maintenance of critical ARG in the K. pneumoniae population.

### Conclusions.

This study demonstrates an independent evolutionary trajectory for CG15 and CG14, marked by expansion of subclades with particular KL types (CG14-KL2 and CG15-KL24/KL112), GyrA and ParC mutations (within CG15), and ARG/MTG in a high diversity of mosaic plasmids. A comprehensive analysis of the plasmidome revealed the contribution of a turbulent flux of plasmids, particularly pervasive F-type and Col plasmids, and adaptive traits (ARGs, MTG, and VFs) to the pangenome, which reflect the exposure to disparate selective pressures of MDR *Enterobacterales*. Finally, this study further highlights the relevance of plasmid data for the understanding of the evolutionary trajectories of ARG and K. pneumoniae subpopulations.

## MATERIALS AND METHODS

### Bacterial isolates and sequencing.

Seven MDR ST15 (3 K24, 1 KL39, 1 KL112, 1 KL19, and 1 KL110) and 2 MDR ST14 (1 KL2 and 1 KL16) isolates were selected for whole-genome sequencing (WGS). These isolates are representative of five CG15 and CG14 lineages previously defined by MLST, PFGE, and FT-IR spectroscopy, circulating as ESBL and/or carbapenemase producers in Portuguese hospitals between 2003 and 2013 (18–21) (Table 1). Bacterial genomic DNA was extracted using the QIAamp DNA minikit (Qiagen), and DNA concentration and purity were measured using a Qubit fluorometer (Life Technologies) and NanoDrop 2000 instrument (Thermo Scientific), respectively. DNA libraries were prepared using the Nextera XT kit (Illumina, San Diego, CA, USA), and 2 × 300-bp paired-end sequence reads with mean coverage of 100× were generated on the MiSeq platform (Illumina). *De novo* assembly was performed with SPAdes v3.9.0 using *k*-mers of 101, 111, 121, and 127 (70), and the quality of the assemblies was evaluated using QUAST (71). The whole-genome shotgun project was deposited in DDBJ/EMBL/GenBank under BioProject accession number PRJNA408270, and assembly statistics concerning the nine isolates sequenced are available in Table 1.

### Publicly available genomes and global data set.

In order to capture CG15 and CG14 diversity and provide robustness to phylogenetic analysis, a total data set of 481 publicly available CG14 (*n* = 110; 1980 to 2018) and CG15 (*n* = 371; 1980 to 2017) K. pneumoniae genomes from NCBI RefSeq (accessed on November 2019) were downloaded, provided they complied with the control quality criteria defined (genome size [4.9 to 6.2 Mb] and GC content [56 to 58%] matching with K. pneumoniae and fewer than 1,000 contigs or *N*_50_ of >20,000) (Table S5 and Fig. S11). Including our 9 genomes, we had an initial data set of 490 K. pneumoniae genomes. Of these, all the genomes without available information concerning the isolation year (*n* = 20) were excluded. Furthermore, 226 genomes (30 CG14 and 196 CG15) showing <21 single nucleotide polymorphisms (SNPs) in the core genome alignment obtained with Roary were discarded since they were epidemiologically related, considering the cutoff proposed by David et al. (11). Furthermore, to evaluate the strength of the temporal signal of our data set and to depict problematic or erroneous sequences (“outliers”), we conducted a linear regression analysis of the root-to-tip genetic distances as a function of the sample collection year, using TempEst v1.5.3 (http://tree.bio.ed.ac.uk/software/tempest/), after which we discarded 9 additional genomes, resulting in 235 deduplicated high-quality genomes.

Our global final data set (*n* = 235) included 65 CG14 (63 ST14 and 2 SLV) and 170 CG15 (162 ST15 and 8 SLV) nonduplicated genomes originated mainly from Europe (55%), Western and South-Eastern Asia (16%), and North America (7%) (Fig. S1). The vast majority were recovered from humans (97%, 75% in the context of infection) and carried genes encoding extended-spectrum β-lactamases, acquired AmpCs, and/or carbapenemases (81%). All the epidemiological and genomic data concerning the final data set of genomes are shown in Table S1.

### Core genome phylogenetic analysis.

To conduct the phylogenetic analyses, the 235 genomes from our final data set were previously annotated using Prokka 1.12 (72) and a core genome alignment was constructed with Roary v3.12 (73) using a blastP identity cutoff of 90% and core genes defined as those being present in more than 90% of the isolates, resulting in a total of 4,420 core genes. Afterwards, 35,783 single-nucleotide variants (SNVs) were extracted from the core genome alignment with SNP-sites (74) and used to construct a maximum likelihood phylogenetic tree with IQ-TREE v1.6.11 (model GTR+F+ASC+R4) rooted with two closely related outgroup genomes based on the study by Wyres et al. (15): K. pneumoniae ST540 isolate Kpn0019 (SRA accession number SRR2098710) and K. pneumoniae ST101 isolate Kp_Goe_33208 (GenBank assembly accession number GCF_001902435.1).

Since CG15 and CG14 evolved separately (Fig. 1) and to improve the reliability of the temporal signal and the inferred predictions, we decided to perform timescaled phylogenetic inference for each CG independently. For this purpose, we generated a recombination-free alignment using Snippy v4.6.0 (https://github.com/tseemann/snippy) and Gubbins v1.12 (75), and we evaluated the strength of the temporal signal for each CG (*R*^2^ for CG14 = 0.1474; *R*^2^ for CG15 = 0.3361) (Fig. S3A and B). The timescaled phylogenetic inference was obtained with BEAST v2.6.2 (run with a Markov chain Monte Carlo length of 1 × 10^9^, sampling every 5 × 10^3^ steps) (76). We used model parameters that had the best fit: GTR substitution model, lognormal relaxed clock, and constant population size. Parameter estimates were computed using Tracer v1.7.1, and a maximum clade credibility tree was obtained with TreeAnnotator v2.6.0 and visualized in FigTree v1.4.4. Additionally, to confirm the significance of our temporal signatures, we conducted a series of date randomization tests for our CG14 data set, running BEAST with true dates in duplicate and in 5 randomized tip date data sets (Fig. S3C).

### MLST/cgMLST and genomic analyses of surface polysaccharides, antimicrobial resistance, virulence, and heavy metal tolerance genes.

MLST and cgMLST were performed using the Institut Pasteur Klebsiella MLST database (https://bigsdb.pasteur.fr/klebsiella/) (6, 13, 17). Genes associated with antimicrobial resistance and virulence (siderophores [yersiniabactin, aerobactin, and salmochelin], colibactin, and regulators of mucoid phenotype [RmpA and RmpA2]) were searched using Kleborate v1.0 (https://github.com/katholt/Kleborate/wiki) (12). Characterization of the capsular locus (KL) and lipopolysaccharide (LPS) O antigen was performed through Kaptive, integrated in Kleborate (12, 77). Additional virulence factors, such as iron uptake systems (*kfu* operon), as well as heavy metal tolerance genes (arsenic [*ars* operon], copper [*pco* cluster], silver/copper [*sil* cluster], tellurite [*ter* operon], and mercury [*mer* operon]) were searched using BIGSdb (https://bigsdb.pasteur.fr/klebsiella/), whereas the *kpi* chaperone-usher pilus system was searched using BLAST (32). Statistical analyses to check the association of the different categorical variables within the clades defined were performed using the χ^2^ test (*P* values of <0.05 were considered statistically significant).

### Accessory genome analysis.

The accessory genome was defined as all genes present in less than 80% of the genomes and was obtained using PATO with default parameters (80% identity and 80% coverage) (30), followed by a detailed study of the protein enrichment in each of the CGs. Accessory gene enrichment analysis according to CG and the respective subclades (KL types) was performed using the function accnet_enrichment_analysis from PATO. This function performs a multihyperparametric test to find overrepresented genes in a cluster in comparison with the general population (i.e., the genome data set). Table S3 lists the genes enriched in the CGs with a *P* value of <0.001 and present in more than 75% of the genomes of the CG, together with their respective annotation. To illustrate the accessory network, we use Gephi for the rearrangement and then we loaded the layout in R to plot the network (Fig. S6A).

The statistical test of accessory genes shared within each CG or within each subclade was performed modeling the distribution of pair sharedness of genes between genomes of each clade/subclade and further correction according to the similarity between genomes. A generalized linear mixed-effects model (lmer R package) was built using the sharedness distribution under a Poisson distribution and corrected with the genomic distance calculated by MASH. The statistical test of the number of accessory genes per genome was performed by nonpaired Student *t* test (stats R package).

### Plasmid identification and analysis.

Plasmids were reconstructed using MOB-recon from MOB-suite software (34). MOB-recon automatically reconstructs sequence plasmids from assembly FASTA files. All 1,050 plasmids predicted and reconstructed by MOB-recon were typed with MOB-typer and then annotated with Prokka (72). A plasmidome was set with PATO by clustering the proteins of each plasmid in protein families of >70% identity. Using the data of presence/absence of each protein family produced by PATO, we built a similarity matrix of Jaccard distances. Then we created a k-nearest neighbor network (K-NNN) with PATO with a parameter of 10 neighbors, allowing reciprocal connections and with a similarity threshold of 0.5 (Jaccard distance) (Table S6). This means that each plasmid is linked to its 10 most similar plasmids as long as they share more than 0.5 Jaccard similarity. In order to remove spurious links, we removed those links in which there was a difference in sequence length between the plasmids of >50% (i.e., the smallest plasmid had to represent at least 50% of the largest plasmid). The plasmid network was arranged by Cytoscape software (78) and imported to R again using the tidygraph R package. Once the network was built, we used the Louvain cluster algorithm (igraph R package). Finally, we incorporated the plasmid typing information (predicted mobility, replicases, and relaxases) to the network. Plasmid distribution over phylogenetic trees was built using ggtree R software. All the data manipulation and visualization were performed using the tidyverse R metapackage.

### Data availability.

The whole-genome shotgun project was deposited in DDBJ/EMBL/GenBank under BioProject accession number PRJNA408270.
