# Supplementary material for: Phylogenomics of Globally Spread Clonal Groups 14 and 15 of Klebsiella pneumoniae
Source: Microbiol Spectr. 2023 Apr 26;11(3):e03395-22. doi: 10.1128/spectrum.03395-22 (PMC10269502; doi:10.1128/spectrum.03395-22)
Supplement: Supplemental file 1 — Fig. S1 to S11. Download spectrum.03395-22-s0001.pdf, PDF file, 4.5 MB [file spectrum.03395-22-s0001.pdf]

Supplementary Material

Phylogenomics of globally spread Clonal Groups 14 and 15 of *Klebsiella pneumoniae*

Carla Rodrigues[1†], Val F. Lanza[2,3], Luísa Peixe[1, 4], Teresa M. Coque[3,5*], Ângela Novais[1,4*], on behalf of the Epidemiological Markers Study Group (ESGEM)

[1] UCIBIO, Applied Molecular Biosciences Unit, Department of Biological Sciences, Laboratory of Microbiology, Faculty of Pharmacy, University of Porto, Porto, Portugal. [†]Current affiliation: Biodiversity & Epidemiology of Bacterial Pathogens, Institut Pasteur, Université Paris Cité, Paris, France.

[2] Unidad de Bioinformática Hospital Universitario Ramón y Cajal (IRYCIS), Madrid, Spain;

[3] CIBER en Enfermedades Infecciosas (CIBERINFEC), Madrid, Spain.

[4]Associate Laboratory i4HB-Institute for Health and Bioeconomy, Faculty of Pharmacy, University of Porto, 4050-313 Porto, Portugal.

[5] Servicio de Microbiología, Hospital Universitario Ramón y Cajal (IRYCIS), Madrid, Spain;

***Corresponding author**

Ângela Novais

E-mail adress: angelasilvanovais@gmail.com**;** aamorim@ff.up.pt

***Co-corresponding author**

Teresa M. Coque

E-mail address: mariateresa.coque@salud.madrid.org; teresacoque@gmail.com

Contents

Figure S1. Worldwide distribution of the main subclades identified within *K. pneumoniae* CG14 and CG15.

Figure S2. Boxplot representing pairwise SNP distances among all the *K. pneumoniae* CG15 and CG14 genomes (A), within CG14 and CG15 clades (B) and within the different main subclades (CG14-I, CG14-II, CG15-IA, CG15-IIA, CG15-IIB) (C).

Figure S3. Temporal analyses in *K. pneumoniae* CG15 and CG15 genomes.

Figure S4. Time-scaled phylogeny of *K. pneumoniae* CG14 genomes and their antimicrobial resistance determinants.

Figure S5. Time-scaled phylogeny of *K. pneumoniae* CG15 genomes and their antimicrobial resistance determinants.

Figure S6. Statistical analysis of the accessory genome of CG14 and CG15 subclades.

Figure S7. K-nearest neighbour network (K-NNN) of *K. pneumoniae* CG14 and CG15 plasmids coloured according to the predicted host-range.

Figure S8. Distribution of the different plasmid groups identified in main CG14 and CG15 *K. pneumoniae* subclades.

Figure S9. Heatmap of the frequency of different antimicrobial resistance genes in the plasmid groups (PGs) identified within *K. pneumoniae* CG14 and CG15 genomes.

Figure S10. Heatmap of the frequency of different metal resistance operons in the plasmid groups (PGs) identified within *K. pneumoniae* CG14 and CG15 genomes.

Figure S11. Phylogenetic structure of the initial dataset analyzed in the study.

Please see separate Excel file for Table S1 to S6.

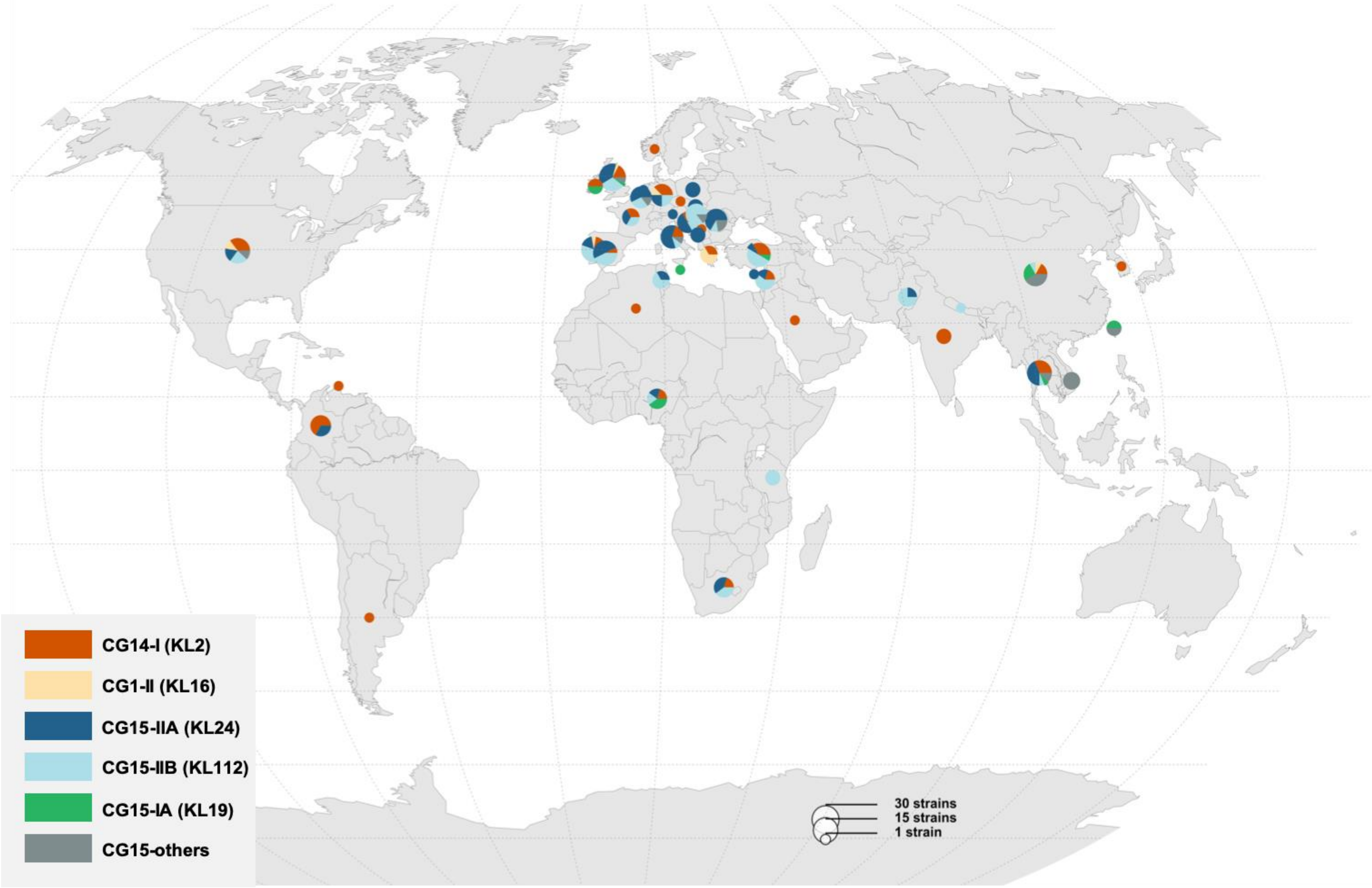

Figure S1. Worldwide distribution of the main subclades identified within *K. pneumoniae* CG14 and CG15.

The pie charts represent the frequency of each clade in each country (see legend).

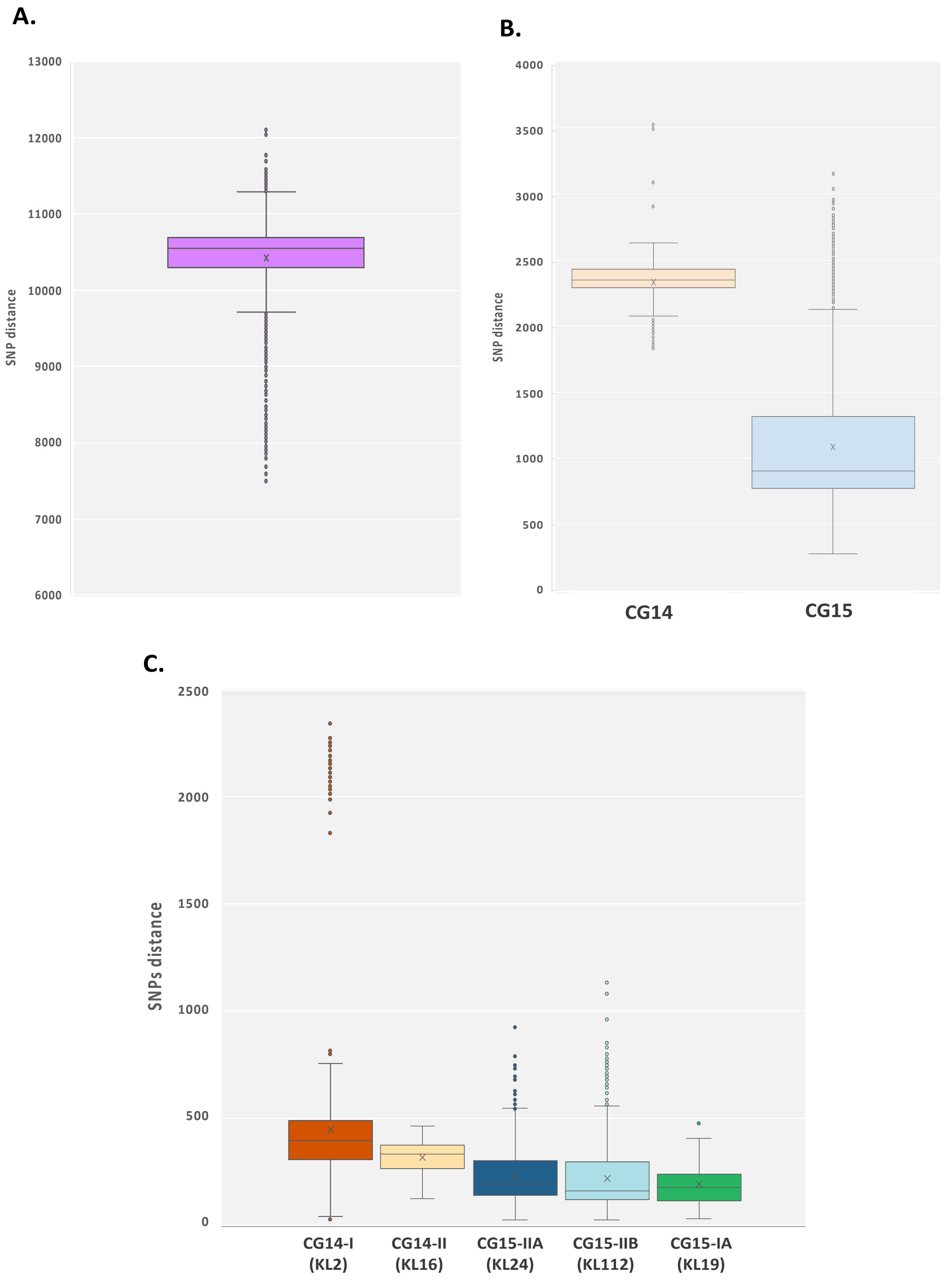

Figure S2. Boxplot representing pairwise SNP distances among all the *K. pneumoniae* CG15 and CG14 genomes (**A**), within CG14 and CG15 clades (**B**) and within the different main subclades (CG14-I, CG14-II, CG15-IA, CG15-IIA, CG15-IIB) (**C**).

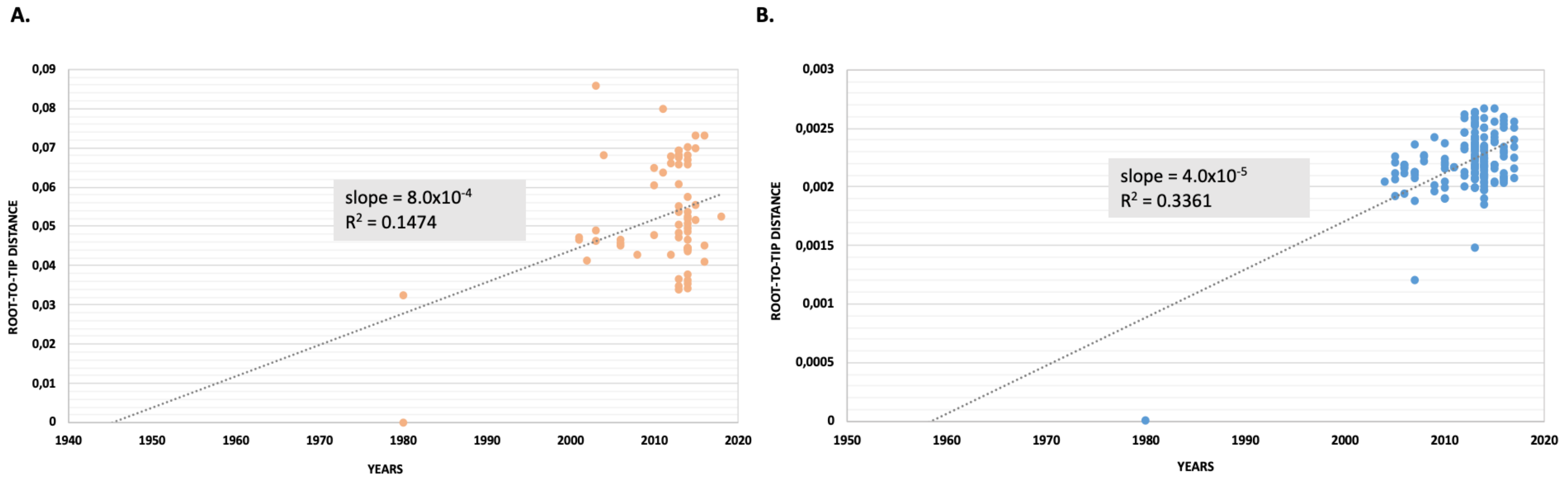

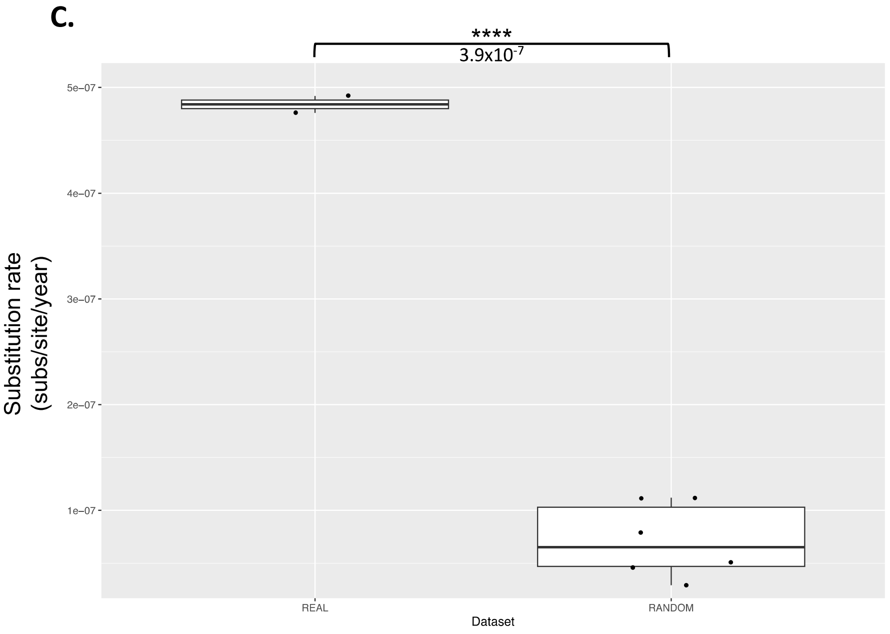

Figure S3. Temporal analyses in *K. pneumoniae* CG15 and CG15 genomes.

A. Linear regression analysis between year of isolation vs root-to-tip distance from the maximum likelihood phylogeny obtained with TempEst for *K. pneumoniae* CG14 genomes; **B**. Linear regression analysis between year of isolation vs root-to-tip distance from the maximum likelihood phylogeny obtained with TempEst for *K. pneumoniae* CG15 genomes; **C**. Bayesian molecular clock estimations based on real dates (run on 2 replicates) and randomized tip dates (6 replicates) for *K. pneumoniae* CG14 dataset. Stars denote statistical significance of $p<0.0001$, assessed using t-test.

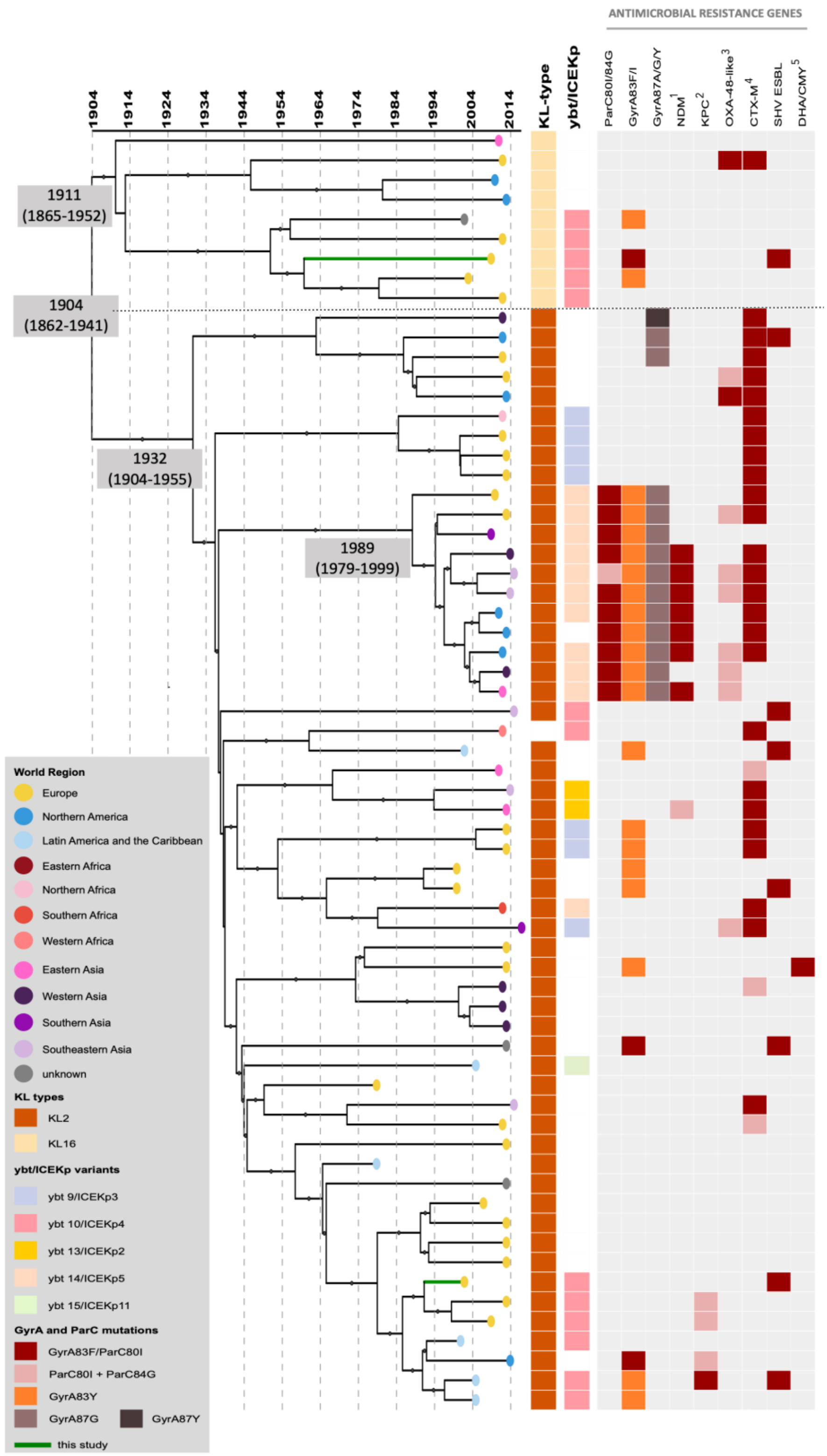

Figure S4. Time-scaled phylogeny of *K. pneumoniae* CG14 genomes and their antimicrobial resistance determinants.

Phylogeny obtained with BEAST. The two main branches correspond to the two main subclades harboring different capsular-types (CG14-I – KL2, CG14-II – KL16). Black dots on main nodes indicate ≥80% posterior probability. Tree tips are colored according to the world region of isolation. KL-type and the yersiniabactin-carrying ICE*Kp* elements are colored in accordance with their variants (see key). The presence of different antimicrobial resistance genes is indicated.

[1], dark pink indicates NDM-1 and light pink indicates other NDM-1 variants; [2], dark pink indicates KPC-2 and light pink indicates KPC-3; [3], dark pink indicates OXA-48 and light pink indicates other OXA-48-like variants; [4], dark pink indicates CTX-M-15 and light pink indicates other CTX-M variants; [5], dark pink indicates DHA-1 whereas light pink stands for CMY.

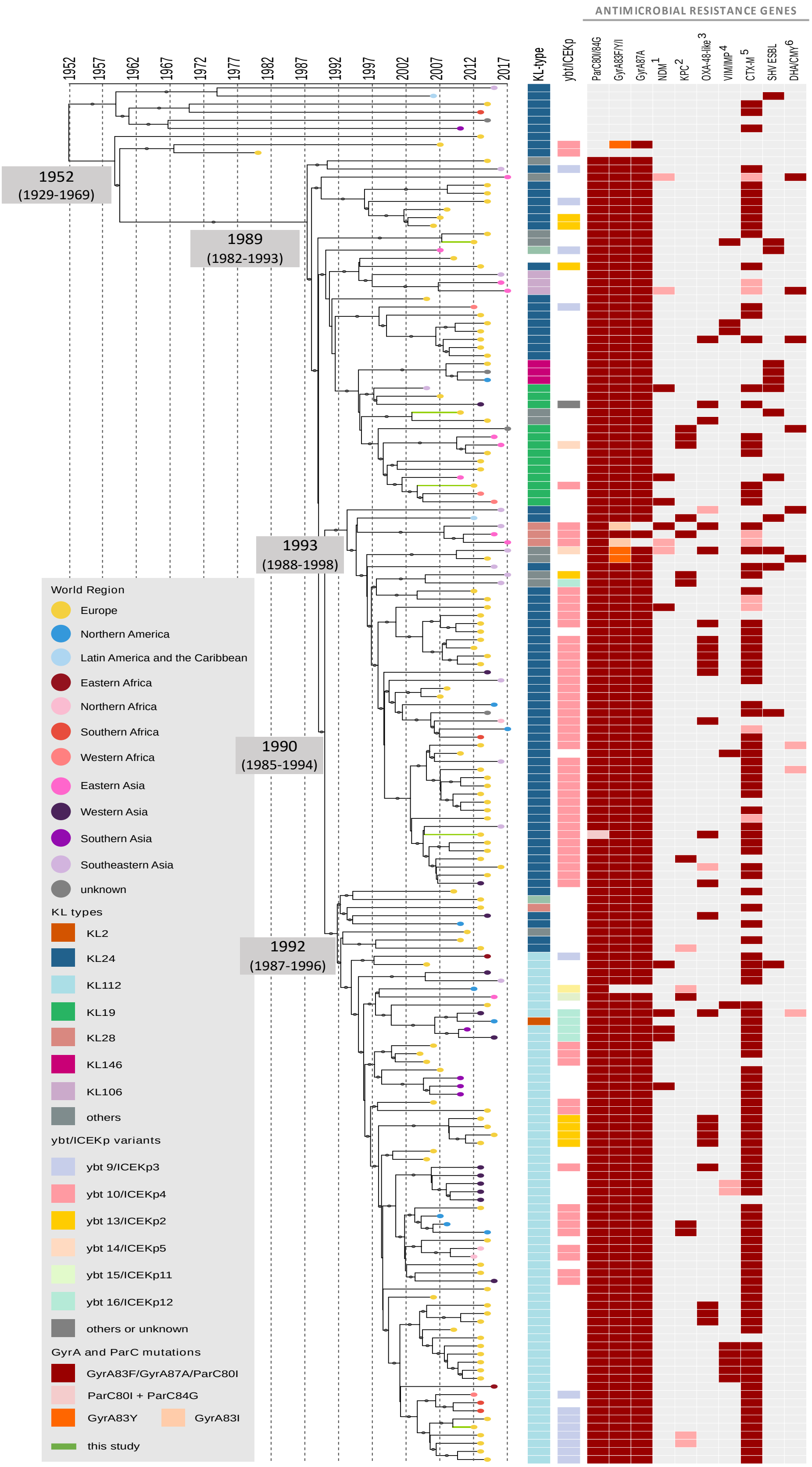

Figure S5. Time-scaled phylogeny of *K. pneumoniae* CG15 genomes and their antimicrobial resistance determinants.

Phylogeny obtained with BEAST. Black dots on main nodes indicate ≥80% posterior probability. Tree tips are colored according to the world region of isolation. KL-type and the yersiniabactin-carrying ICE*Kp* elements are colored in accordance with their variants (see key). The presence of different antimicrobial resistance genes is indicated.

[1], dark pink indicates NDM-1 and light pink indicates other NDM-1 variants; [2], dark pink indicates KPC-2 and light pink indicates KPC-3; [3], dark pink indicates OXA-48 and light pink indicates other OXA-48-like variants; 4, dark pink indicates VIM enzymes whereas light pink indicates IMP enzymes; [5], dark pink indicates CTX-M-15 and light pink indicates other CTX-M variants; [6], dark pink indicates DHA-1 whereas light pink stands for CMY.

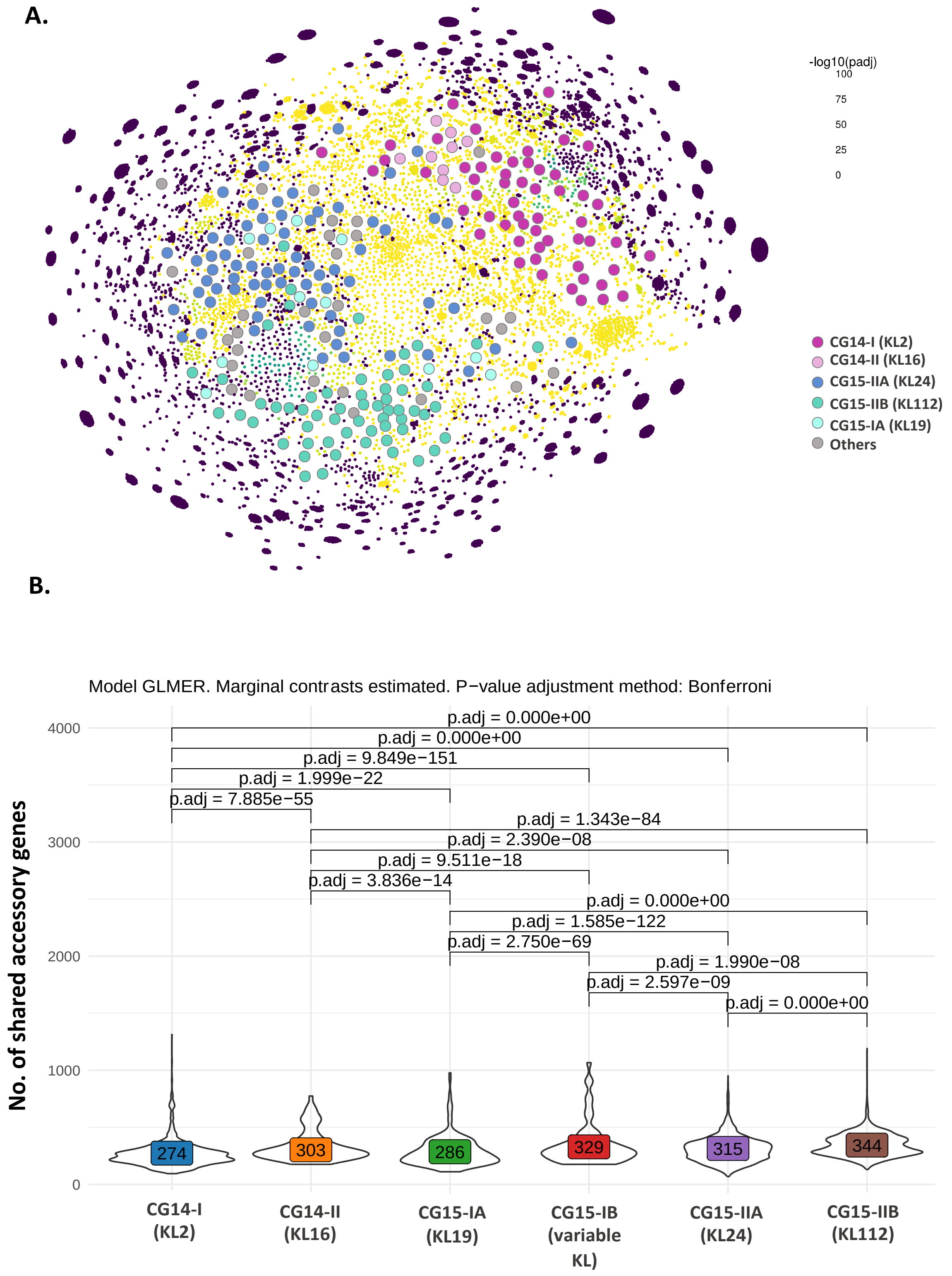

Figure S6. Statistical analysis of the accessory genome of CG14 and CG15 subclades**.**

Accessory genes shared within main CG14 or CG15 subclades. Each set of shared genes between each genome was evaluated by correcting the model by the genomic distance between the strains in order to

mitigate the bias produced by sampling bias. The distribution was modelled with a GLME and a Poisson distribution.

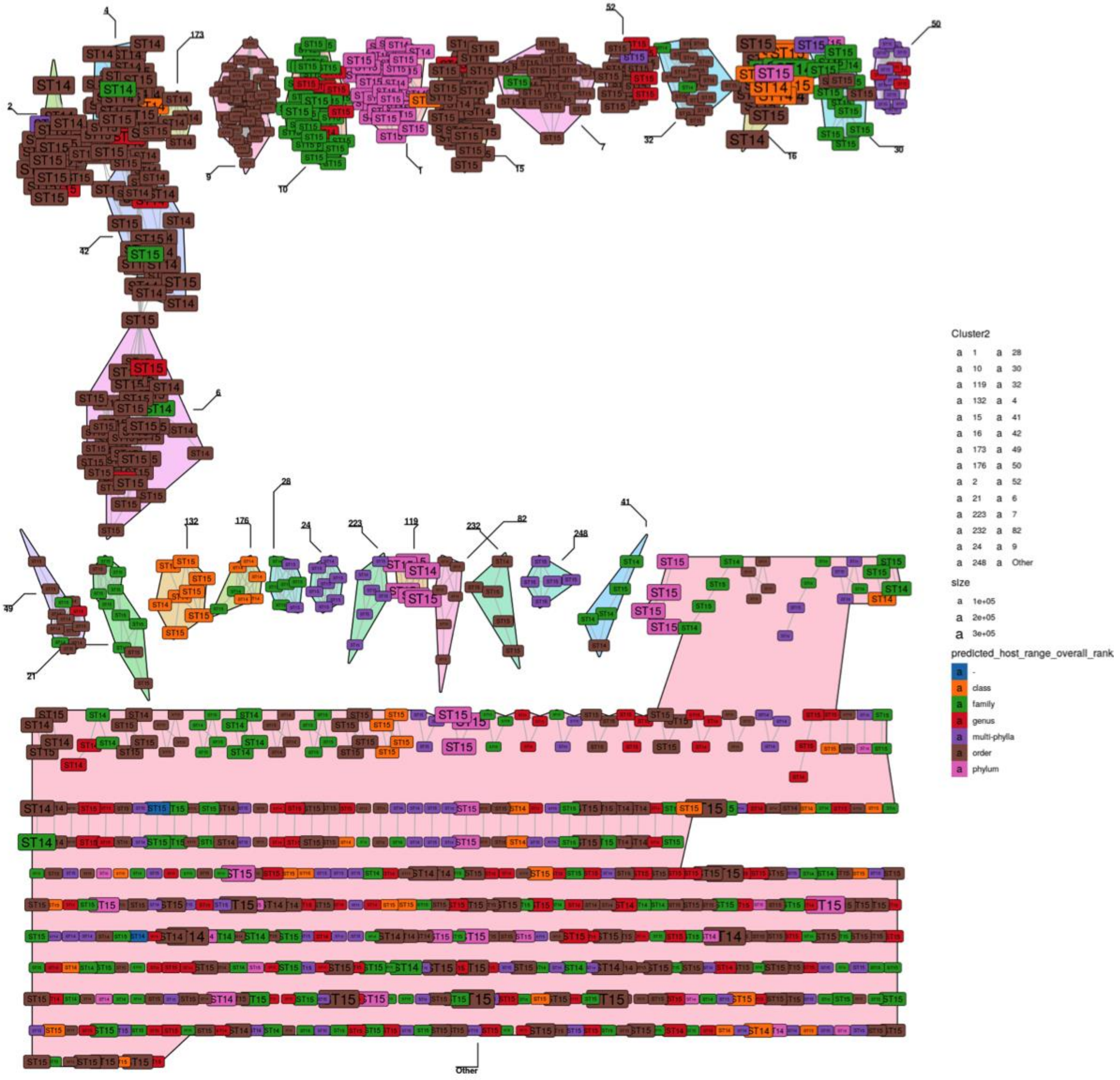

Figure S7. K-nearest neighbour network (K-NNN) of *K. pneumoniae* CG14 and CG15 plasmids coloured according to the predicted host-range.

Network was built using the PATO k-nnn function. Each node represents a plasmid and is colored according to the host-range. Each plasmid is connected with the 10 best hits if their Jaccard similarity is at least 0.5. Plasmid clusters are defined using the Louvain algorithm over the network structure.

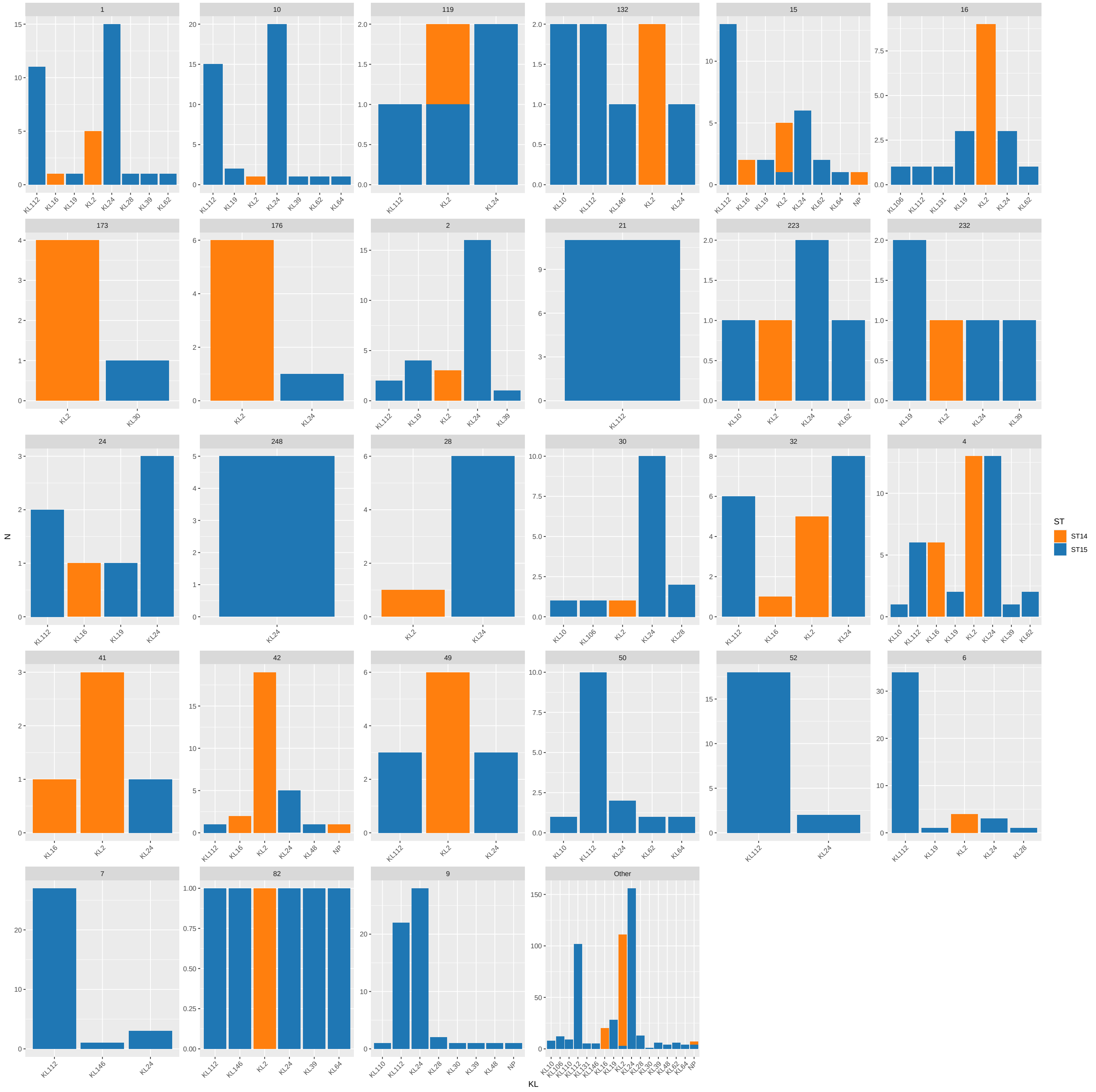

Figure S8. Distribution of the different plasmid groups identified in main CG14 and CG15 *K. pneumoniae* by capsular-type.

N, number of genomes, KL, capsular-type

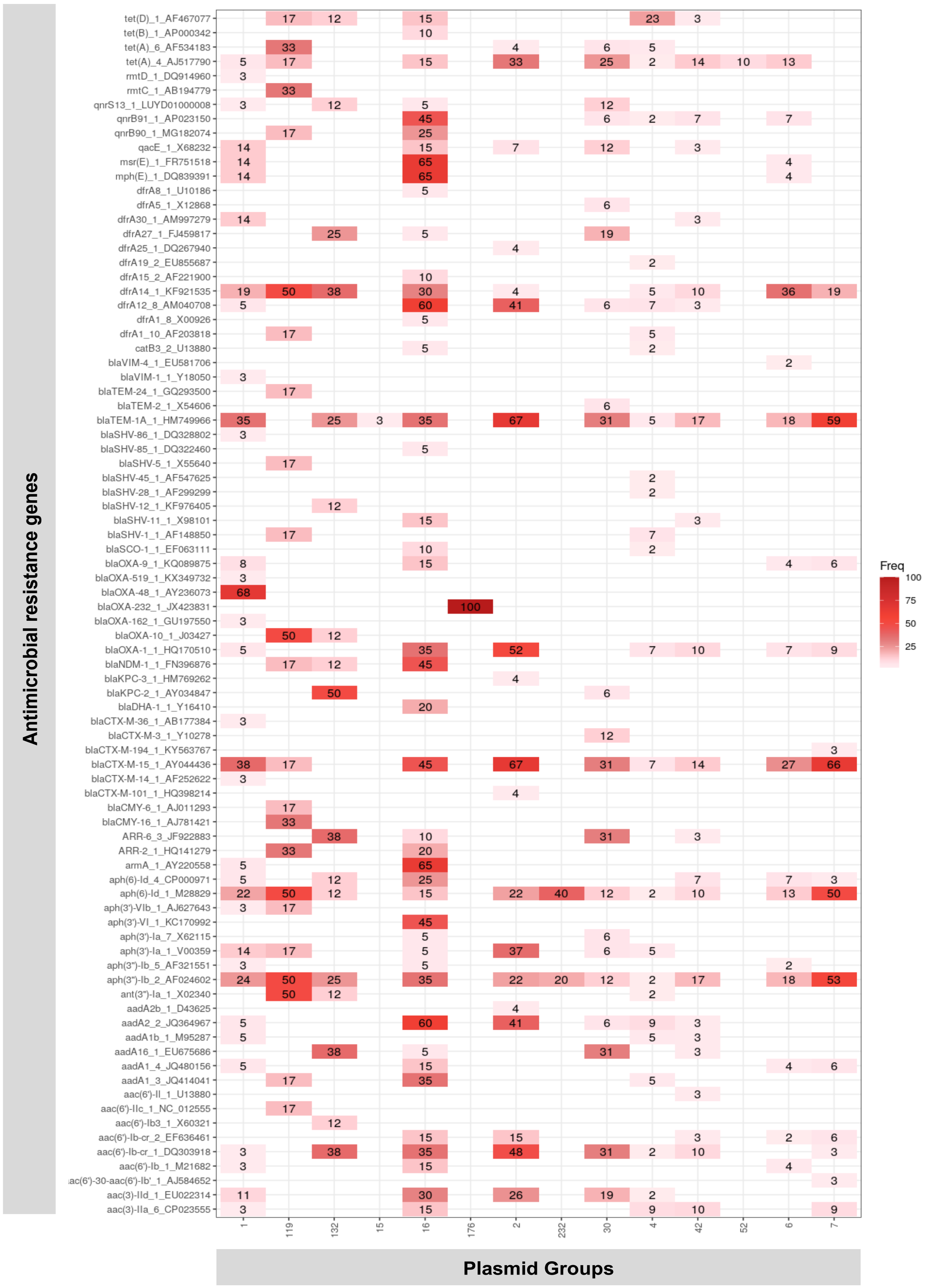

Figure S9. Heatmap of the frequency of different antimicrobial resistance genes in the plasmid groups identified within *K. pneumoniae* CG14 and CG15 genomes.

The proportion of AMR genes identified in each plasmid group is indicated in each square.

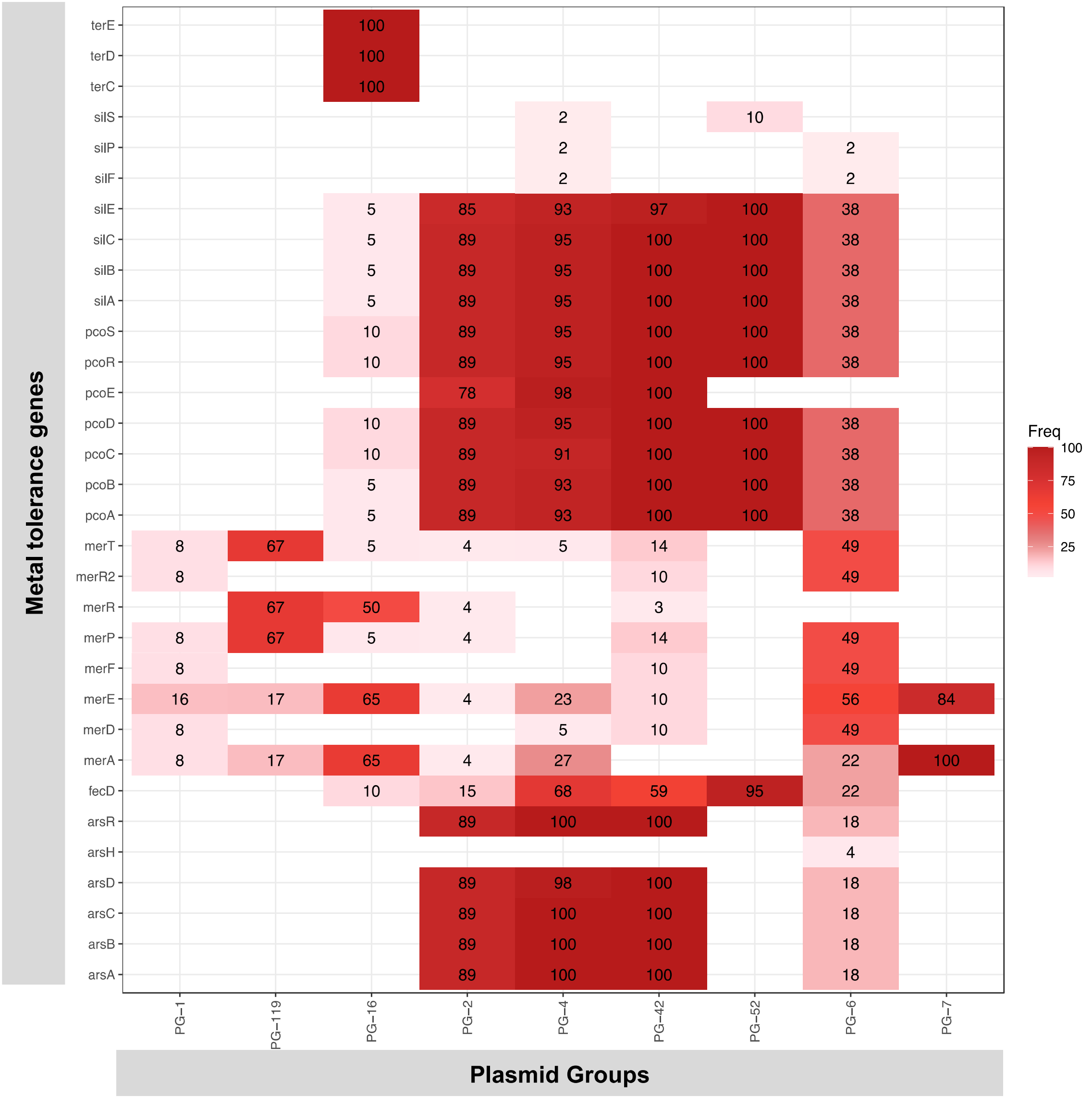

Figure S10. Heatmap of the frequency of different metal resistance operons in the plasmid groups (PGs) identified within *K. pneumoniae* CG14 and CG15 genomes.

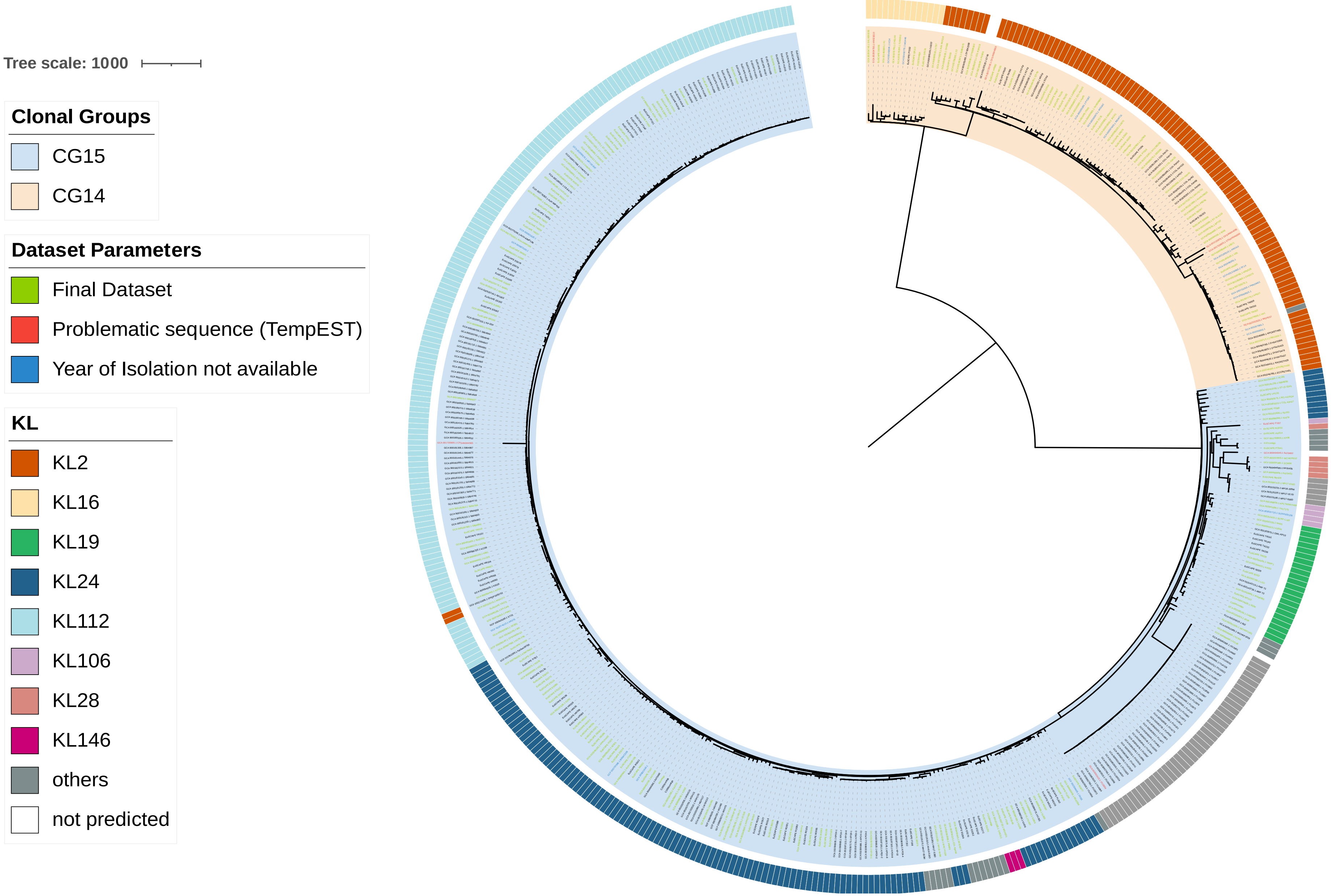

Figure S11. Phylogenetic structure of the initial dataset (n=490) analyzed in the study.

The tree was obtained by maximum likelihood analysis based on the final alignment of concatenated nucleotide sequence alignments of 4,420 core genes. *Kp* ST540 Kpn0019 (accession number: SRR2098710) and a *Kp* ST101 Kp_Goe_33208 (GCF_001902435.1) were used to root the tree. Main capsular-types (KL) identified are indicated. In green the final genome dataset (n=235).
